# Muscle-specific miR-499-5p delivered by small extracellular vesicles impairs endothelial function and ischemic hindlimb recovery in diabetic mice

**DOI:** 10.1186/s12933-025-02825-2

**Published:** 2025-07-10

**Authors:** Zhongjian Cheng, May M. Truongcao, Vandana Mallaredy, Maria Cimini, Charan Thej, Darukeshwara Joladarashi, Carolina Gonzalez, Cindy Benedict, Suresh K. Verma, Venkata Naga Srikanth Garikipati, Raj Kishore

**Affiliations:** 1 Aging + Cardiovascular Discovery Center, Lewis Katz School of Medicine, Temple University, 3500 Broad Street, Philadelphia, PA 19140 USA; 2 Department of Cardiovascular Sciences, Lewis Katz School of Medicine, Temple University, 3500 Broad Street, Philadelphia, PA 19140 USA; 3 Department of Medicine-Cardiovascular Disease, The University of Alabama at Birmingham, 703 19th South Street, Birmingham, AL 35233 USA

**Keywords:** Diabetes, Ischemic hindlimb, miR-499-5p, Endothelial cell dysfunction, Skeletal muscle derived-small extracellular vesicles

## Abstract

**Background:**

Emerging evidence suggests that skeletal muscle cells (SKMC) play critical roles in the defective angiogenic response in diabetic critical limb ischemia. However, the molecular mechanisms linking skeletal muscle to impaired angiogenic properties of endothelial cells (EC) remain unidentified. The current study investigates how muscle-specific miR-499-5p may impair EC function in diabetic ischemic limbs.

**Methods:**

Eight-week-old, male C57BL/6 J, db/ + and db/db mice were employed. Hind limb ischemia was established by unilateral ligation of the left femoral artery, and blood flow recovery was monitored using Laser Doppler perfusion imaging (LDPI). ECs and SKMCs were isolated from sham or ischemic hind limbs (IHL). SKMC-derived small extracellular vesicles (SKMC-sEVs) were isolated from the culture medium of SKMCs by ultra-centrifugation.

**Results:**

miR-499-5p level was markedly increased in SKMCs and unexpectedly in ECs from hindlimb of db/db mice. Ischemic injury further enhanced miR-499-5p levels in ECs from IHL of db/db mice. Angiogenic activity was reduced in ECs from IHL of db/db mice and in miR-499-5p-overexpressing ECs. Intramuscular injection of lentiviral-anti-miR-499-5p improved blood perfusion and angiogenesis in IHL of db/db mice. Mechanistically, we found that diabetic SKMC sEVs carried high levels of miR-499-5p and transferred miR-499-5p to ECs. Intramuscular injection of diabetic SKMC-sEVs repressed IHL recovery in wildtype mice. Blocking sEV biosynthesis/release by GW4869 markedly improved neovascularization and blood perfusion in IHL of db/db mice. We identified that SRY (Sex-Determining Region Y)-Box 6 (SOX6) is a direct downstream target of miR-499-5p. Silencing of SOX6 suppressed release of proangiogenic factors from ECs. Targeted reduction of miR-499-5p significantly enhanced SOX6 levels in ECs from IHL of db/db mice. Finally, overexpression of SOX6 improved the angiogenic property of ECs from IHL of db/db mice.

**Conclusions:**

SKMC-sEV-mediated transfer of myo-miR-499-5p and subsequent suppression of SOX6 plays a critical role in diabetes-impaired neovascularization in IHL of db/db mice. Targeting miR-499-5p-mediated pathogenic communication between SKMCs and ECs may be a novel therapeutic avenue for critical limb ischemia in diabetic patients.

**Graphical abstract:**

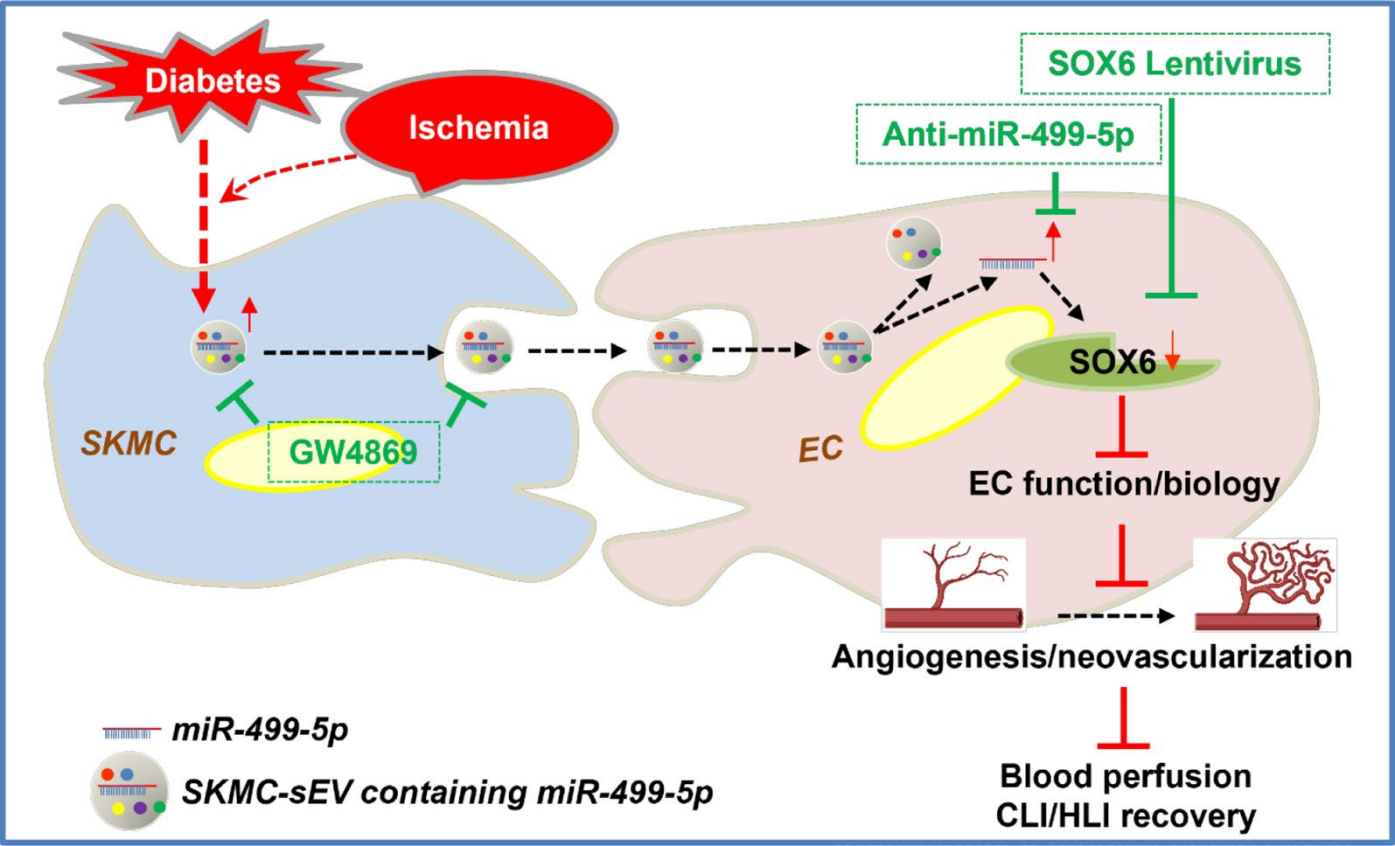

**Supplementary Information:**

The online version contains supplementary material available at 10.1186/s12933-025-02825-2.

## Research insights

**What is currently know about this topic?**

- CLI is one of the leading causes of mortality in diabetic patients.
- Improving angiogenesis thus increasing blood flow is considered as one promising strategy for therapeutics of CLI.
- EC is the essential component of angiogenesis. The underlying mechanisms of EC dysfunction in CLI of diabetic patients remain unclear.

**What is the key research question?**

What is the underlying mechanism of EC dysfunction in diabetic CLI?

**What is new?**

- Expression of miR-499-5p, a muscle-specific miR, is increased in ECs from hindlimb of db/db mice which is further upregulated by ischemic injury.
- SKMC-sEVs transfer miR-499-5p to ECs. Inhibition of sEV release or silencing of miR-499-5p improves angiogenesis and blood flow in IHL of db/db mice.
- miR-499-5p impairs EC function in IHL of db/db mice via, at least partially, SOX6.

**How might this study influence clinical practice?**

- Strategies inhibiting abnormal SKMC-sEV production may have potential benefit against CLI injury in diabetic patients.
- Local anti-miR-499-5p and SOX6 overexpression therapy may potentially rescue diabetes induced suppression of angiogenesis in CLI of diabetic patients.
- sEV-carried miR-499-5p may be a biomarker for severity and prognosis of CLI in diabetic patients.

## Introduction

Critical limb ischemia (CLI) is one of the leading causes of mortality in patients with diabetes, a disease affecting 38 million people in the United States. CLI is defined by obstruction of the lower limb arteries, resulting in ischemic conditions in the lower limbs, which can lead to tissue damage, necrosis, amputation and even mortality. Incidence of CLI in patients with diabetes is extremely high (up to 76% in some studies) [1]. Disease severity at the time of presentation and progression of CLI in diabetic patients has also been noticed to be worse with a 5–7 times higher amputation risk compared to that in diabetic patients [2, 3].

Although diabetic CLI has been extensively studied, none of the available treatment options provide significant clinical benefits. Improving angiogenesis/neovascularization to improve blood perfusion and oxygen/nutrient supply in ischemic limbs is a promising strategy for effective treatment of CLI. Unfortunately, treatments ranging from pro-angiogenic growth factors administration to cell therapy, and gene therapy in CLI patients have failed to translate success from animal studies to the clinic; thus, therapeutic angiogenesis remains a major challenge. Moreover, diabetes disrupts the intrinsic angiogenic potential which creates a exacerbates obstacle in applying therapeutic angiogenesis for CLI in diabetic patients [4]. Better understanding of the molecular mechanisms of diabetes-impaired angiogenesis in ischemic limbs, is required to identify novel effective therapeutics for ischemic limb injury in patients with diabetes.

Accumulating evidence suggests that endothelial cells (ECs), an essential component for angiogenesis, play a critical role in the process of neovascularization in response to ischemic stimuli. We have reported that diabetes impairs EC function and disrupts the intrinsic angiogenic potential of ECs [5–10]. However, the underlying mechanisms of diabetes-induced EC dysfunction remain incompletely understood. Recently, the most abundant cell type in what context? In the body? In the extremities? In the limbs? and most proximal to ECs, skeletal muscle cells (SKMCs) have become attractive targets for therapeutic angiogenesis of CLI/IHL [11, 12]. SKMCs secrete angiogenic factors and promote cell–cell communication between SKMC and EC, either through direct cell–cell contact, transfer of secreted paracrine molecules, or exchange of extracellular vesicles (EVs) [13]. Enhancing the paracrine function of healthy SKMCs is an effective potential therapeutic strategy for ischemic hindlimbs in experimental animals [12, 14]. Whether aberrant SKMC-derived signals alter the phenotype of ECs, rendering EC refractive to therapeutic angiogenesis in CLI/IHL diabetes models, remains unclear.

microRNAs (miRs) are bioactive small non-coding RNAs containing 21–23 nucleotides which interact with complementary sequences in the 3’untranslated region (3’UTR) of protein-coding mRNAs, resulting in inhibition of protein translation and/or mRNA degradation [15]. Many miRs have been reported to be involved in the pathogenesis of cardiovascular diseases by regulation of mRNA/protein levels of downstream targets. We have reported that miRs play a critical role in impairment of angiogenesis and in ischemic tissue injury recovery after heart failure [16, 17]. Some miRs are not ubiquitously expressed, but are expressed in a tissue/cell specific manner [18]. miR-499 is a muscle-specific miRNA (designated myomiR) encoded by intron 19 of *myh7b*―a sarcomeric myosin gene expressed in skeletal muscle. miR-499 plays a crucial role in regulating muscle fiber type, promoting slow-twitch fiber formation and contributing to muscle metabolism and function [19]. Emerging evidence indicates that miR-499 (miR-499-3p and miR-499-5p) level is modulated by diabetes. miR-499 level was increased in heart, retinal tissue and cells from streptozotocin (STZ)-induced diabetic rats [20, 21]. In diabetes, enhanced miR-499 expression inhibits proliferation and promotes apoptosis of retinal cells, and contributes to hepatic insulin resistance [21, 22]. Several studies also report that overexpressing miR-499 interferes with EC function [23, 24]. However, whether and how myomiR-499 impairs EC function in ischemic limbs in diabetes has never been studied.

Small extracellular vesicles (sEVs), also called exosomes, are nano-sized, lipid bilayer membrane nanovesicles (diameter: 30 to 200 nm) secreted by cells into the extracellular space in response to pathophysiological stimuli. sEVs are important mediators of intercellular and extracellular communication by facilitating exchange of genetic material, such as DNA fragments, mRNAs and miRs [25]. We have shown that circulating sEVs play a critical role in the pathogenesis of ischemic hearts and hindlimbs in experimental animal models [6, 26–28]. Whether diabetes alters the expression of the myomiR-499 in SKMC-derived sEVs and whether these sEVs directly transfer miR-499 to ECs and induced EC dysfunction is not known.

Utilizing extensive in vitro mechanistic studies and a surgically induced hindlimb ischemia model in diabetic mice in vivo, we provide evidence that SKMC-sEVs deliver muscle-specific miR-499-5p to ECs, thus impair the angiogenic properties of ECs and reduce blood perfusion in IHL of diabetic mice. Further, we identify SOX6 as a direct target of miR-499 and provide evidence that anti-miR-499 therapy enhances ischemic limb angiogenesis in diabetic mice. Targeting miR-499-5p-mediated pathological communication between SKMCs and ECs may be a novel strategy for attenuating diabetic exacerbation of ischemic limb injury.

## Methods

All animal experiments adhered to the National Institutes of Health Guidelines on the Use of Laboratory Animals and were approved by the Institutional Animal Care and Use Committee of Temple University. Materials, experimental procedures, data collection protocols, and analytic methods will be made available to other researchers as requested for purposes of experiment reproduction, procedure replication, and for collaborative study. A detailed description of all materials and methods can be found in the Supplemental Materials.

### Statistical analysis

Data are reported as Mean ± SEM. For analysis of differences between two groups, unpaired student’s t test was performed. For multiple groups with one independent variable, data were analyzed by one-way analysis of variance (ANOVA) followed by Tukey’s multiple comparisons test. For multiple groups with two independent variables, data were analyzed by a two-way analysis of variance (ANOVA) followed by Tukey’s multiple comparisons test. For all statistical tests, *p* values less than 0.05 were considered statistically significant. All statistical analyses were performed via GraphPad Prism 9 software.

## Results

### miR-499-5p levels are increased in SKMCs and ECs from hindlimb of db/db mice and are further upregulated after ischemic injury

**Fig. 1:**
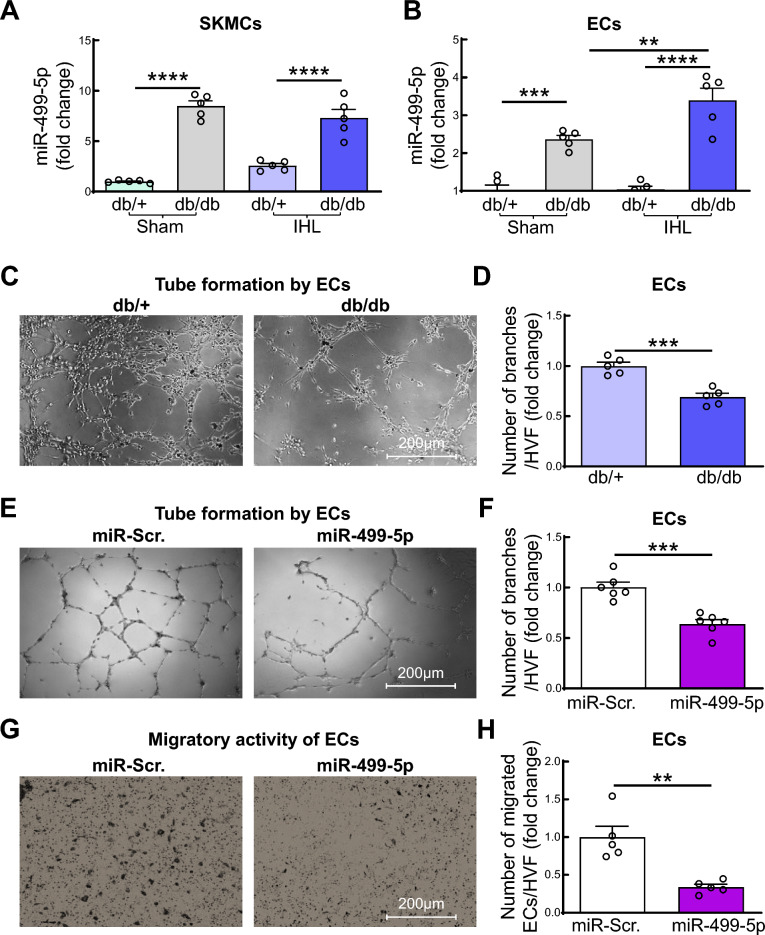
Diabetes induces muscle-specific miR-499-5p expression in SKMC and EC. **A–B** miR-499-5p levels in mouse SKMCs (A) and ECs (B). Mouse primary SKMCs and ECs were isolated from sham or IHL of 8–10-week, male db/ + and db/db mice 3 days post-surgery. n = 5, one-way ANOVA, Tukey multiple comparisons test. **p < 0.01; ***p < 0.001; ***p < 0.0001). **C–D** Representative microphotographs (**C**) and quantification of tube formation (**D**) by ECs. Mouse primary ECs were isolated from IHL of 8–10-week, male db/ + and db/db mice 3 days post-surgery. n = 5, unpaired student *t* test, ***p < 0.001. **E–F** Representative microphotographs (**E**) and quantification of tube formation (**F**) by ECs; **G–H** Representative microphotographs (**G**) and quantification (**H**) of migratory activity of ECs. HMVECs were transfected with miR-499-5p mimics (20 ng/ml) or scramble (Scr.) for 48 h. n = 5–6, unpaired student *t* test. **p < 0.01; ***p < 0.001. HVF: high-power visual field

As miR-499 is a skeletal-muscle-enriched, muscle-specific miRNA, encoded by intron 19 of *myh7b* (a sarcomeric myosin gene expressed in skeletal muscle) [29], we first examined miR-499-3p and -5p levels in primary SKMCs isolated from sham-operated and ischemic hindlimbs of db/ + and db/db mice, on day 3 post-HLI surgery. SKMCs were isolated by digesting hindlimb skeletal muscles with collagenase 1 and cells were identified by immuno-staining with anti-desmin antibody as previously described [1]. More than 95% of isolated SKMCs were viable and desmin positive (Fig. S1). Compared to control db/ + mice, in db/db mice the expression of miR-499-5p was dramatically increased in SKMCs both at base line (8.5-fold) and after ischemia (7.32-fold, Fig. 1A). Ischemic injury increased miR-499-5p levels in SKMCs of db/ + mice but did not reach statistical significance (*p* = 0.165). We also observed that miR-499-5p levels were markedly enhanced (3.48-fold) in hindlimb tissues of unmanipulated db/db mice (Fig. S2A) and in high fat diet (HFD)-induced diabetic mice (Fig. S2B-D). Whereas, miR-499-3p expression in SKMCs was barely detected and not affected by diabetes and/or ischemic injury (data not shown). Therefore, in the present study, we focused on the role of miR-499-5p in the pathogenesis of diabetes/ischemia-induced EC dysfunction in IHL of db/db mice.

Next, we examined miR-499-5p level in ECs which were isolated from hindlimbs of sham and IHL of db/ + and db/db mice 3 days post-surgery by using collagenase I and CD31 Dynabeads as described previously [5–7]. We observed that miR-499-5p was significantly increased in ECs from sham hindlimb of db/db mice compared to those from db/ + mice (Fig. 1B). Of note, ischemic injury significantly upregulated diabetes-enhanced miR-499-5p expression in ECs of db/db mice (Fig. 1B).

To obtain direct evidence that ECs from IHL of db/db mice are functionally impaired, we examined tube formation capacity by primary ECs isolated from IHL of db/db mice and db/ + mice 3 days post-surgery. A tube formation assay was conducted using the primary ECs (Fig. S3) after 2 weeks in culture. We are the first to provide evidence that tube formation by primary ECs from IHL of db/db mice is significantly impaired compared to that from IHL of db/ + mice (Fig. 1C, D).

### Overexpression of miR-499-5p impairs tube formation and migratory activity of ECs

To study the direct role of miR-499-5p on EC function, we examined tube formation and migratory activity of human microvascular endothelial cells (HMVECs) with overexpression of miR-499-5p mimics. In accordance with previous studies that overexpression of miR-499 impairs the angiogenic property of ECs [23, 24], we found that tube formation and migratory activity were significantly impaired in HMVECs that overexpressed miR-499-5p (Fig. 1E-H). These results suggest that enhanced miR-499-5p expression may play a critical role in EC dysfunction in IHL of db/db mice.

### Treatment with anti-miR-499-5p improves blood perfusion, capillary and arterial density in IHL of db/db mice

**Fig. 2:**
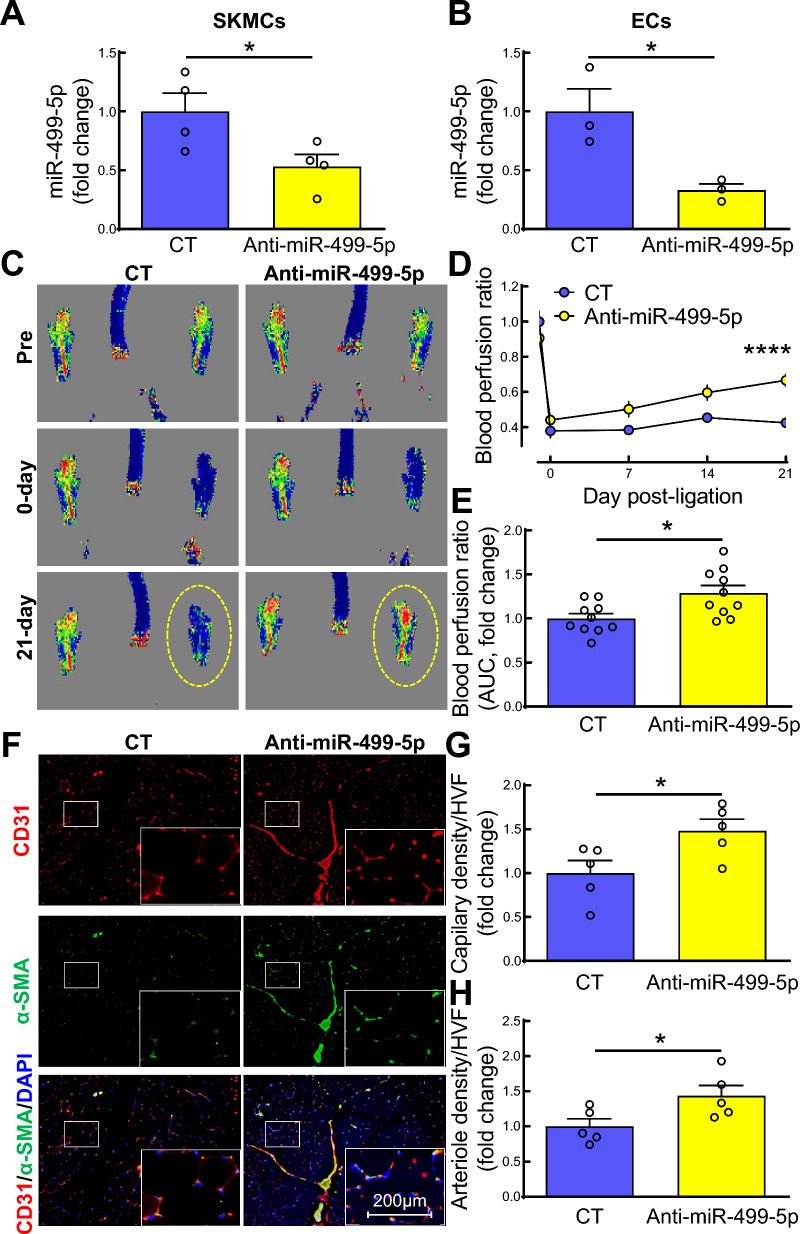
Silencing of miR-499-5p improves blood perfusion and vessel density in IHL of db/db mice. Unilateral HLI surgery was established by ligation of left femoral artery 8–10-week-old, male db/db mice. Control (CT) or anti-miR-499-5p lentivirus was administered immediately by intramuscular injection after ligation of left femoral artery (5*10^7^ IU in 100 µl 1xPBS/mouse, at five injection sites, three sites in medial thigh and two sites in gastrocnemius muscles, respectively. 20 µl/per site). Doppler imaging of blood flow was taken pre-, and at day 0, 7, 14 and 21 post surgery. On day 21 post-surgery, the gastrocnemius muscles from IHL of db/ + and db/db mice were dissected for isolation of SKMCs and ECs, and immunostaining. **A–B** miR-499-5p levels in SKMCs (A) and ECs (B). n = 3–4, unpaired student *t* test. *p* < 0.05; **C–D** Representative photographs of Doppler imaging of blood flow (C) and quantification of blood perfusion on IHL presented as ratio of IHL/non-IHL on indicated time point (D). n = 10, two-way ANOVA, Tukey multiple comparisons test. ****p < 0.0001; **E** Quantification of blood perfusion on IHL presented as area under carve (AUC) of (D). n = 10, unpaired student *t* test, *p < 0.05; **F** Representative microphotographs of immunostaining with CD31 (EC marker, red), α-smooth muscle actin (α-SMA, smooth muscle marker, green) and 4′,6-diamidino-2-phenylindole (DAPI, nuclear marker, blue) in sections from gastrocnemius muscles on IHL; **G-H** Quantification of capillary density (CD31^+^, G) and arterial density (CD31^+^/α-SMA^+^, H) in sections from gastrocnemius muscles on IHL. n = 5, unpaired student *t* test. *p < 0.05. HVF: high-power visual field

To obtain direct evidence that miR-499-5p participates in EC dysfunction in IHL of db/db mice, we examined effects of miR-499-5p inhibition on blood perfusion, capillary, and arterial density in IHL of db/db mice db/db mice were administered anti-miR-499 lentivirus by intramuscular injection (anti-miR-499-5p, miRa-off-mmu-miR-499-5p lentivirus, Applied Biological Materials, LV002-c-53399, 5 × 10^7^ IU/mouse) immediately after ligation of the left femoral artery. db/db mice injected with the same dose of control lentivirus (CT, ABM, m002) served as controls. We observed that intramuscular injection of anti-miR-499-5p lentivirus resulted in significantly decreased miR-499-5p levels in both SKMCs (Fig. 2A) and ECs (Fig. 2B) from IHL of db/db mice 21 days post-surgery. Silencing of miR-499-5p significantly improved blood perfusion in IHL of db/db mice (Fig. 2C-E). We also observed that targeting miR-499-5p expression significantly improved capillary (CD31^+^/α-SMA^−^) and arteriole (CD31^+^/α-SMA^+^) density in IHL of db/db mice (Fig. 2F-H). These data provide convincing evidence that miR-499-5p expression plays a critical role in impaired blood perfusion and neovascularization in IHL of db/db mice.

### Hyperglycemia upregulates miR-499-5p levels in SKMCs and SKMC-derived sEVs

**Fig. 3:**
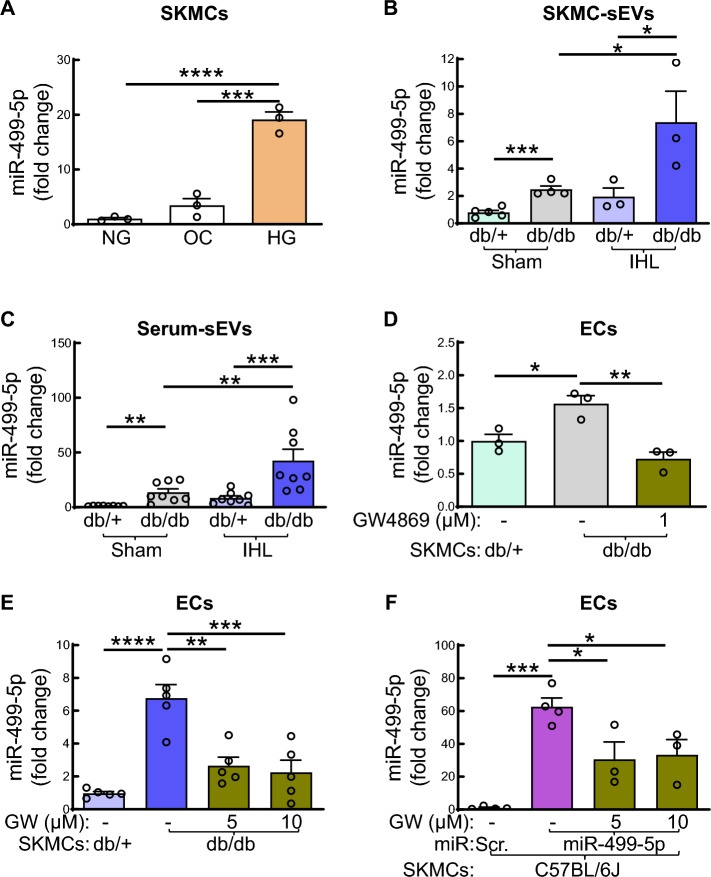
Diabetic SKMC-sEVs transfer miR-499-5p from SKMCs to ECs. **A** miR-499-5p levels in wildtype SKMCs. SKMCs isolated from C57BL/6 J mice were cultured in corresponding culture medium with additional 25 mM D-glucose (high glucose. HG) for 48 h after 6 h. starvation (1% FBS). Cells cultured with 0 mM additional D-glucose (normal glucose, 5 mM D-glucose, NG) or 25 mM mannitol served as HG and osmotic control (5 mM D-glucose, OC), respectively. **B-C** miR-499-5p levels in mouse SKMC-sEVs (B) and serum-sEVs (C). SKMC-sEVs and serum-sEVs were isolated from sham or IHL of 8–10-week, male db/ + and db/db mice 3 days post-surgery. **D** miR-499 levels in ECs co-cultured with SKMCs from sham hindlimbs of db/ + and db/db mice (at age of 8–10-week) in the presence or absence of GW (1 µM) for 48 h. (upper chamber). HMVECs were cultured in the lower chamber. n = 3, one-way ANOVA, Tukey multiple comparisons test. *p < 0.05; **p < 0.01. **E** miR-499 levels in ECs co-cultured with SKMCs from IHL of db/ + and db/db mice (3 days post-IHL, at age of 8–10-week) in the presence or absence of GW (5 or 10 µM) for 48 h. (upper chamber). HMVECs were cultured in the lower chamber. n = 5, one-way ANOVA, Tukey multiple comparisons test. **p < 0.01; ***p < 0.001; ****p < 0.0001. **F** miR-499 levels in ECs co-cultured with miR-499-5p-overexpressed SKMCs. SKMCs isolated from hindlimbs of C57BL/6 J mice were transfected with miR-499-5p mimics or miR scramble (Scr.) for 72 h., then cultured in the upper channel in the presence or absence of GW (5 or 10 µM) for 48 h. HMVECs were cultured in the lower chamber. n = 3–5, one-way ANOVA, Tukey multiple comparisons test. *p < 0.05; ***p < 0.001. GW: GW4869

Because SKMC is the cellular source of miR-499-5p in vivo, [29] we examined effects of hyperglycemia, the key pathogenic condition in diabetes, on miR-499-5p levels in SKMCs. Primary SKMCs were isolated from hindlimbs of wild-type C57BL/6 J mice and cultured in medium with an additional 25 mM D-Glu (high glucose, HG) for 48 h after starvation in 1% FBS for 6 h. Cells cultured in culture medium without additional D-glucose (normal glucose, NG) or with additional 25 mM mannitol (osmotic control, OC) served as controls. High glucose markedly increased miR-499-5p levels in SKMCs compared to SKMCs cultured under NG or OC conditions (Fig. 3A).

### SKMC-sEVs from IHL of db/db mice carry higher level of miR-499-5p

sEVs are important mediators of cell–cell communication by facilitating exchange of genetic material including miRs [25]. To study whether skeletal muscle-derived sEVs (SKMC-sEVs) are responsible for the presence of diabetes- and/or ischemic injury-induced miR-499-5p in ECs, we examined miR-499-5p levels in SKMC-sEVs in sham-operated and IHL db/ + and db/db mice. SKMCs were isolated from sham and IHL of db/ + and db/db mice 3 days post-surgery and cultured in exosome-free FBS under either NG (SKMCs from db/ + mice) or HG condition (SKMCs from db/db mice). Then the SKMC-sEVs were isolated from the culture medium by ultra-centrifugation as we described previously (Fig. S4) [6, 28]. We found that SKMC-sEVs from sham hindlimbs of db/db mice carried significantly higher levels of miR-499-5p compared with that from db/ + mice (Fig. 3B). Ischemic injury significantly increased miR-499-5p level in SKMC-sEVs from db/db mice. Moreover, we also examined miR-499-5p levels in serum sEVs (serum-sEVs) and found that there was a remarkable increase in miR-499-5p levels in serum-sEVs of db/db mice compared to that in db/ + mice (Fig. 3C). Ischemic injury further enhanced miR-499-5p levels in serum-sEVs of db/db mice (Fig. 3C).

### SKMC-sEVs transfer miR-499-5p from SKMCs to ECs

To study whether miR-499 expression in EC in diabetic mice is mediated by SKMC-sEVs, we examined miR-499-5p levele in HMVECs co-cultured with SKMCs in a Boyden chamber in the presence or absence of sEV inhibitor GW4869 as previously described [30]. The SKMCs were isolated from sham or IHL of db/ + and db/db mice or hindlimbs of C57BL/6 J mice (wild-type), then cultured for 7–10 days under either NG (SKMCs from db/ + and C57BL/6 J mice) or HG condition (SKMCs from db/db mice), respectively. Following the glucose-adjusted culture period, t SKMCs were seeded on the upper chamber of a Boyden chamber in the presence or absence of sEV inhibitor GW4869 (Fig. S5) for 1 h. After 1 h, HMVECs were seeded in the lower chamber of the Boyden chamber. We found that miR-499-5p levels were markedly increased in ECs co-cultured with SKMCs from sham/IHL of db/db mice for 48 h compared to that with SKMCs from db/ + mice (Fig. 3D, E). Inhibition of SKMC-sEV release from diabetic and/or ischemic SKMCs by GW4869 significantly reduced miR-499-5p levels in ECs (Fig. 3D, E). Moreover, overexpression of miR-499-5p in wild-type SKMCs increased miR-499-5p level in co-cultured ECs, which conversely was significantly reduced by GW4869 (Fig. 3F). Our findings indicate that SKMC-sEVs transfer miR-499-5p to ECs in diabetes/ischemic conditions.

### Injection of diabetic SKMC-sEVs impairs blood perfusion recovery and neovascularization in IHL of wildtype mice

**Fig. 4:**
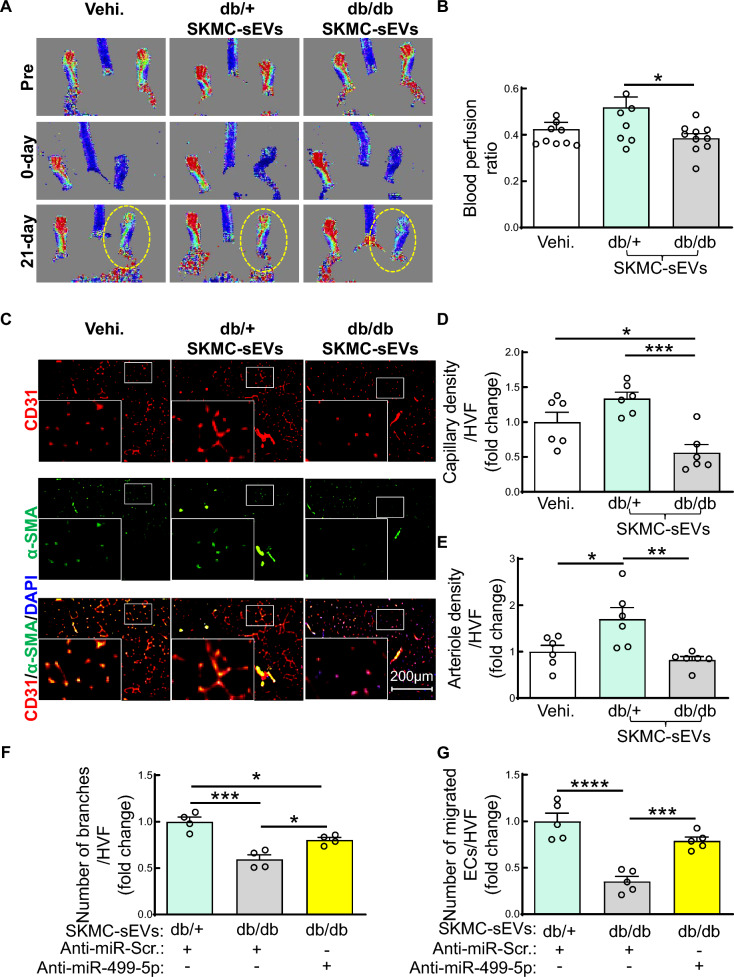
Diabetic SKMC-sEVs impair ischemic limb blood flow recovery and angiogenesis in non-diabetic mice. SKMC-sEVs were collected from cultured medium of SKMCs isolated from hindlimbs of 8–10-week-old, male db/ + or db/db mice. Male, 8–10-week-old C57BL/6 J mice were conducted with IHL surgery by ligation of left femoral artery and administered with diabetic or non-diabetic SKMC-sEVs immediately after ligation of left femoral artery by intramuscular injection (3*10^9^ particles in 100 µl 1xPBS/mouse, at five injection sites, three sites in medial thigh and two sites in gastrocnemius muscles, respectively. 20 µl/per site). Mice received equal volume of saline (vehicle, Vehi.) served as controls. Doppler imaging of blood flow was taken at pre-, and day 0 and 21 post-IHL. **A–B** Representative photographs of doppler imaging of blood flow (A) and quantification of blood perfusion (presented as ratio of IHL/non-IHL, B) on IHL at 21-day post-surgery. n = 10, one-way ANOVA, Tukey multiple comparisons test. *p < 0.05; **C** Representative microphotographs of immunostaining of CD31 (EC marker, red), α-smooth muscle actin (α-SMA, smooth muscle marker, green) and 4′,6-diamidino-2-phenylindole (DAPI, nuclear marker, blue) in sections from gastrocnemius muscles on IHL; **D–E** Quantification of capillary (CD31^+^, D) and arterial (CD31^+^/α-SMA^+^, E) density. n = 6, one-way ANOVA, Tukey multiple comparisons test. *p < 0.05; **p < 0.01; ***p < 0.001. **F–G** Quantification of tube formation (F) and migratory activity (G) of ECs. HMVECs were transfected with anti-miR-499-5p (50 ng/ml) for 72 h. then treated with SKMC-sEVs from db/db mice (10^8^ particles/ml) for 48 h. before tube formation and migratory activity assay. HMVECs treated with SKMC-sEVs from db/ + and anti-miR-Scr. served as controls. n = 4– 5, one-way ANOVA, Tukey multiple comparisons test. *p < 0.05; ***p < 0.001; ****p < 0.0001. HVF: high-power visual field

To further provide direct evidence that SKMC-sEVs play a critical role in diabetes-impaired IHL recovery, we examined effects of diabetic SKMC-sEVs on IHL recovery in wildtype mice. SKMC-sEVs were isolated from culture medium of SKMCs isolated from hindlimbs of db/db or db/ + mice. Male C57BL/6 J mice were subjected to HLI surgery by ligation of the left femoral artery and administered diabetic or non-diabetic SKMC-sEVs immediately after artery ligation by intramuscular injection (3*10^9^ particles/mouse) as we described previously [5, 6]. We observed that intramuscular injection of diabetic SKMC-sEVs significantly suppressed blood perfusion in IHL of wildtype mice compared to recipients injected with either saline or non-diabetic SKMC-sEVs 21 days post-IHL (Fig. 4A, B). Diabetic SKMC-sEVs also markedly reduced capillary (CD31^+^) and arteriole (CD31^+^/α-SMA^+^) density in IHL of wildtype mice compared to those injected with injection of either saline (vehicle, Vehi) or non-diabetic SKMC-sEVs (Fig. 4C, E). Our findings indicate that impaired EC function in IHL of db/db mice is, at least partially, dependent upon SKMC-sEV-delivered miR-499-5p.

### Knockdown of miR-499-5p rescues diabetic SKMC-sEV-impaired tube formation and migratory activity of ECs

To evaluate the role of miR-499-5p in diabetic SKMC-sEV-impaired EC function, we examined if inhibiting miR-499-5p in ECs could rescue diabetic SKMC-sEV-impaired EC function. HMVECs were transfected with anti-miR-499-5p for 72 h. then treated with SKMC-sEVs from culture medium of SKMCs isolated from hindlimb of db/db mice (10^9^ particles/ml) for 48 h. before tube formation and migratory activity assays. Silencing of miR-499-5p in HMVECs significantly prevented diabetic SKMC-sEV-mediated repression of tube formation and migratory activity (Fig. 4F-G). These findings further confirm that miR-499-5p plays a critical role in diabetic SKMC-sEV-impaired EC function.

### Systemic blocking of sEV biosynthesis/release in vivo markedly improves IHL recovery in db/db mice

**Fig. 5:**
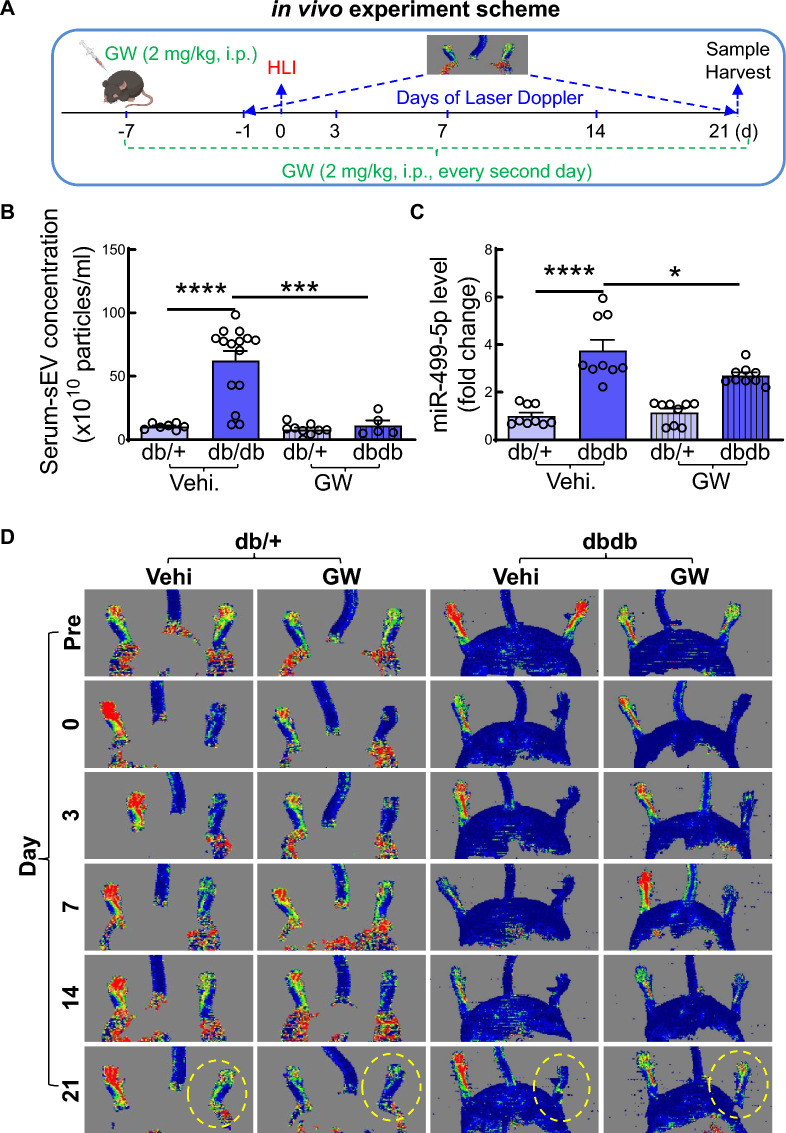

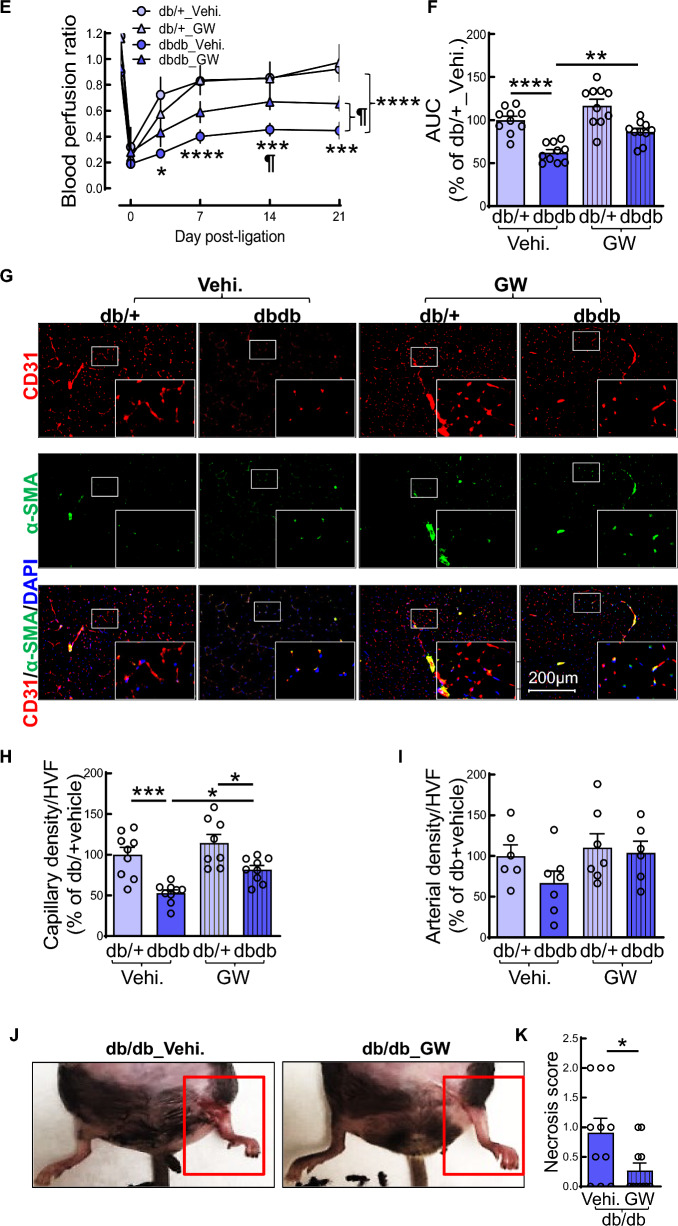
Systemic inhibition of sEV release improves IHL injury recovery in db/db mice. GW was administered 7 days before IHL/sham surgery in 8–10-week-old, male db/ + and db/db mice by intraparietal injection (2 mg/kg BW, every second day, diluted by 1XPBS containing 0.005% DMSO). Mice injected with equal volume of 1XPBS containing 0.005% DMSO (vehicle, Vehi) served as controls. Doppler imaging was taken at pre-, and day- 0, 3, 7, 14 and 21 post-IHL. In the end of experiment, serum-sEVs were collected; ECs were isolated from gastrocnemius muscles on IHL; Narcosis score was calculated. **A** In vivo experiment scheme; **B** Serum-sEV concentrations. n = 5– 15, one-way ANOVA, Tukey multiple comparisons test. ***p < 0.001; ****p < 0.0001;** C** miR-499-5p levels in ECs. n = 9, one-way ANOVA, Tukey multiple comparisons test. ***p < 0.001; ****p < 0.0001;** D** Representative photographs of doppler imaging of blood flow; **E** Quantification of blood perfusion (presented as ratio of IHL/non-IHL) on IHL at pre-, day-0, 3, 7, 14 and 21 post-IHL; n = 10, two-way ANOVA, Tukey multiple comparisons test. *p < 0.05 vs db/ + _Vehi; ***p < 0.001 vs db/ + _Vehi; ****p < 0.0001 vs db/ + _Vehi; *p*^¶^ < 0.05 vs db/db-GW; **F** Quantification of blood perfusion on IHL is presented as area under carve (AUC) of ratio of IHL/non-IHL. n = 10, one-way ANOVA, Tukey multiple comparisons test. **p < 0.01; ****p < 0.0001; **G** Representative microphotographs of immunostaining of CD31 (EC marker, red), α-smooth muscle actin (α-SMA, smooth muscle marker, green) and 4′,6-diamidino-2-phenylindole (DAPI, nuclear marker, blue) in sections from gastrocnemius muscles on IHL; **H–I** Quantification of capillary density (CD31^+^, H) and arterial density (CD31^+^/α-SMA^+^, I). n = 6– 9, one-way ANOVA, Tukey multiple comparisons test. *p < 0.05; ***p < 0.001; **J** Representative photographs of narcosis; **K** Quantification of narcosis score. n = 11, unpaired student *t* test. *p* < 0.05. GW: GW4869; Vehi: Vehicle

To confirm the role of sEV-derived-miR-499-5p in diabetes-impaired IHL recovery, we evaluated if systemic blocking of sEV biosynthesis/secretion by intraperitoneal injection of GW4869 can improve IHL recovery in db/db mice. GW4869 is a pharmacological compound that inhibits sEV/exosome biogenesis/secretion. [31] GW4869 was administered 7 days before IHL surgery and continued up to 21 days post-IHL (2 mg/kg, every second day, Fig. 5A) as described previously [31]. We observed that GW4869 dramatically reduced serum-sEV concentration in db/db mice (Fig. 5B). Strikingly, administration of GW4869 significantly reduced miR-499-5p levels in ECs from IHL of db/db mice (Fig. 5C). Moreover, we observed that systemic blocking of sEV biosynthesis/release significantly improved blood perfusion (Fig. 5D-F), increased capillary density (Fig. 5G-I), and prevented necrosis (scored as described previously [32], Fig. 5J-K) of IHL of db/db mice. These results indicate that SKMC-sEVs are, at least partially, responsible for enhanced miR-499-5p levels in EC and impair IHL recovery in db/db mice.

### miR-499-5p targets transcription factor SOX6 in ECs

**Fig. 6:**
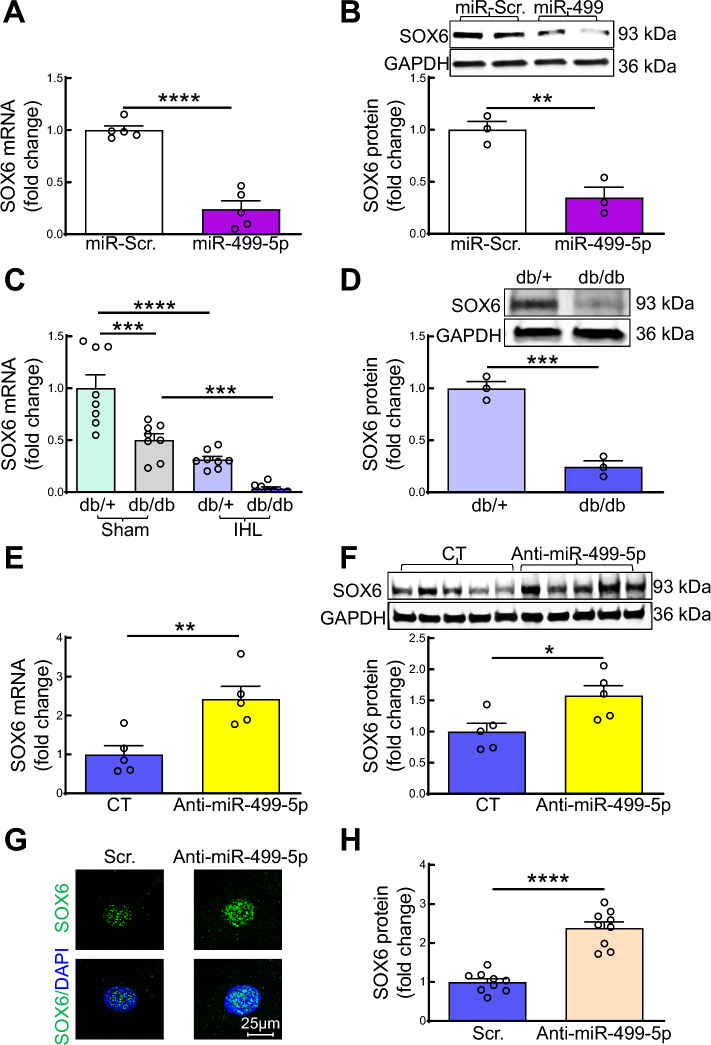
miR-499-5p targets transcription factor SOX6 in EC. **A–B** SOX-6 mRNA (A) and protein (B) levels in ECs. HMVECs were transfected with miR-scramble (Scr.) or miR-499-5p (20 ng/ml) for 72 h. n = 3– 5, unpaired student *t* test. **p < 0.01; ****p < 0.0001. **C-D** SOX-6 mRNA (C) and protein levels in ECs from db/ + and db/db mice. Mouse primary ECs were isolated from sham or IHL of 8–10-week, male db/ + and db/db mice 3 days post-surgery. n = 3 + 8, one-way ANOVA, Tukey multiple comparisons test (mRNA) and unpaired student *t* test (protein). ***p < 0.001; ****p < 0.0001. **E–F** SOX-6 mRNA (E) and protein (F) levels in ECs from IHL db/db mice with or without administration of anti-miR-499-5p lentivirus. IHL surgery was conducted by ligation of left femoral artery 8–10-week-old, male db/db mice. Control (CT) or anti-miR-499-5p lentivirus (5*10^7^ IU in 100 µl 1xPBS/mouse, at five injection sites, three sites in medial thigh and two sites in gastrocnemius muscles, respectively. 20 µl/per site) was administered immediately by intramuscular injection after ligation of left femoral artery. Mouse primary ECs were isolated from IHL of db/db mice at 21-day post IHL- surgery. n = 5, unpaired student *t* test (protein). *p < 0.05; **p < 0.01. **G** Representative microphotographs of immunostaining of SOX6 (green) and 4′,6-diamidino-2-phenylindole (DAPI, nuclear marker, blue) in ECs from IHL of db/db mice; **H** Quantification of SOX6 density in nuclei of ECs from IHL of db/db mice. Mouse primary ECs were isolated from IHL of db/db mice 3 days post-HLI and cultured for 14 days then transfected with anti-miR-499-5p or anti-miR-scramble oligonucleotides (Scr., 50 ng/ml for 72 h.) before the immunostaining. n = 9, unpaired student *t* test. ****p < 0.0001. HVF: high-power visual field

In silico analysis from miR target prediction programs including MiRanda, TargetScan, and Mirtarget identified SOX6, a member of the sex-determining region Y-box (SOX) transcription factor family, as one of the most likely candidates for a pivotal miR-499-5p target gene due to multiple miR-499-5p binding sites in both human and murine SOX6 3’UTR (Fig. S6). In accordance with the in silico analysis, overexpression of miR-499-5p mimics in HMVECs significantly reduced SOX6 mRNA expression compared to HMVECs treated with a scrambled miR control (Fig. 6A). The decrease in SOX6 mRNA levels was concomitant with a decrease in SOX6 protein levels (Fig. 6B). Our findings suggest that miR-499-5p suppresses SOX6 expression in ECs. We also observed that SOX6 mRNA level was significantly decreased in ECs from hindlimb of db/db mice compared with that from db/ + mice (Fig. 6C). Ischemic injury markedly reduced SOX6 mRNA and protein expression in ECs from db/ + and db/db mice (Fig. 6C, D). We also observed that targeting miR-499-5p by intramuscular injection of anti-miR-499-5p lentivirus markedly restored SOX6 mRNA and protein expression in ECs from IHL of db/db mice (Fig. 6E, F). Targeting miR-499-5p by transfection of miR-499-5p antagomir significantly increased SOX6 expression in nucleus of the diabetic ECs from IHL of db/db mice (Fig. 6G, H). Our findings strongly suggest that miR-499-5p downregulates SOX6 in diabetic ECs thereby impairing EC function.

### SOX6 knockdown recapitulates miR-499-5p anti-angiogenic effects in ECs

**Fig. 7:**
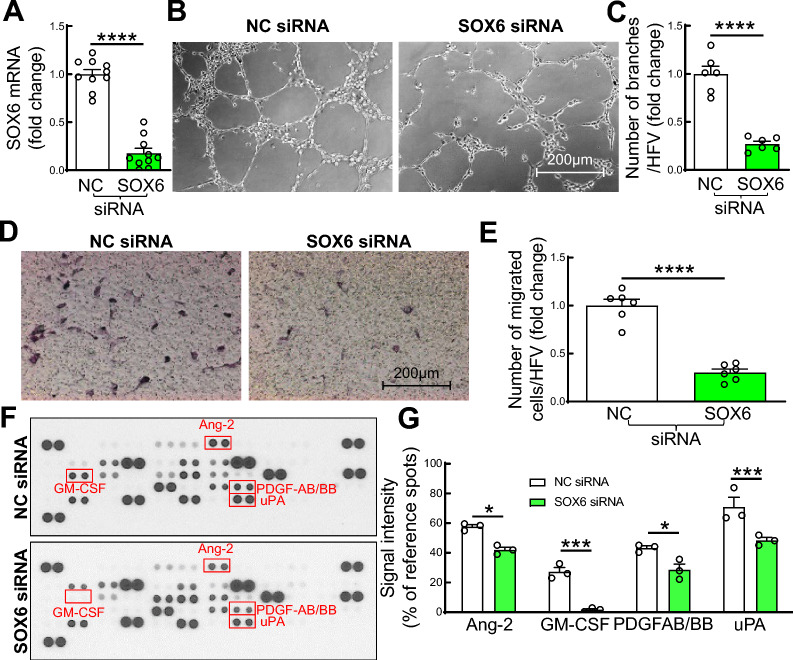
SOX6 knockdown impairs tube formation, migratory activity and pro-angiogenic factor release from ECs. **A** SOX6 mRNA levels in ECs; **B–C** Representative microphotographs (B) and quantification of tube formation (C) by ECs; **D–E** Representative microphotographs (D) and quantification of migratory activity of ECs; **F–G** Representative photographs of angiogenic protein profile array (F) and quantification of spot densities from the membranes of angiogenic protein profile array (G) with ECs culture media. HMVECs were transfected with either SOX6 siRNA or negative control siRNA (NC, 200 nM) for 72 h. n = 3– 10, unpaired student *t* test, *p < 0.05; **p < 0.001; ****p < 0.0001. Ang-2: angiopoietin-2; GM-CSF: granulocyte–macrophage colony-stimulating factor; PDGF-AB/BB: platelet-derived growth factor AB/BB; uPA: urokinase-type plasminogen activator

Although several members of the SOX family have been reported to improve the angiogenic property of ECs [33–35], the role of SOX6 in EC function has not been reported. To investigate the role of SOX6 in regulation of EC function, we examined tube formation and migratory activities of EC following knockdown of SOX6. HMVECs were transfected with SOX6 siRNA (200 nM) or negative control siRNA (NC) for 72 h. We observed that SOX6 mRNA levels were dramatically decreased in ECs transfected with SOX6 siRNA (Fig. 7A). Knockdown of SOX6 in ECs impaired both tube formation and migration of ECs compared to those transfected with NC siRNA (Fig. 7B–E). By angiogenic protein profile array, we observed that silencing of SOX6 in HMVECs significantly decreased secretion of several pro-angiogenic factors, including angiopoietin-2 (Ang-2), granulocyte–macrophage colony-stimulating factor (GM-CSF), platelet-derived growth factor AB/BB (PDGF-AB/BB) and urokinase-type plasminogen activator (uPA) from conditioned culture medium of HMVECs transfected with SOX6 siRNA (Fig. 7F–G). Our findings suggest that miR-499-5p impairs EC function in diabetes by SOX6-regulated suppression of pro-angiogenic factors. Whether SOX6 binds to the promoters of Ang-2, GM-CSF, AB/BB, and/or uPA thus regulating their expression in diabetic ECs is unclear. Which SOX6-regulated pro-angiogenic factors play a critical role in impaired EC function/angiogenesis in diabetes remains unclear.

### Overexpression of SOX6 improves the angiogenic property of primary ECs from IHL of db/db mice

**Fig. 8:**
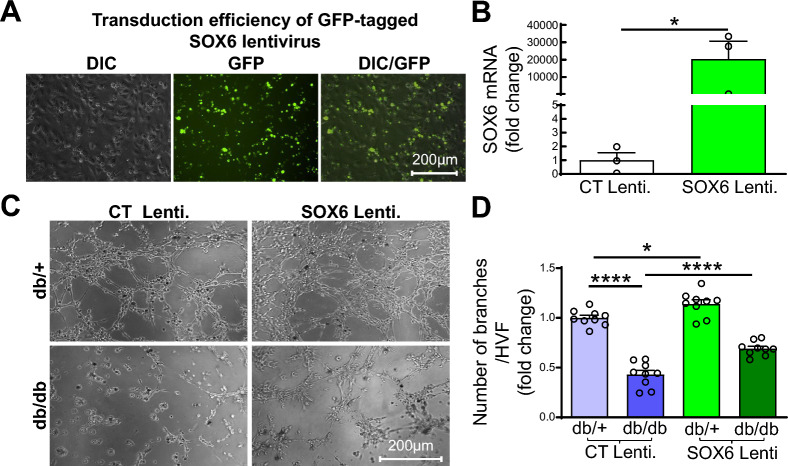
Overexpression of SOX6 improves diabetic EC functions. Mouse primary ECs were isolated from IHL of db and db/db mice 3 days post-IHL, cultured for 14 days, then transduced with GFP-tagged SOX6 or control lentivirus (CT, 10^7^ IU/ml) for 72 h. **A** Representative microphotographs of GFP-tagged lentivirus-transduced ECs; **B** SOX6 mRNA levels in ECs. n = 3, unpaired student *t* test. *p < 0.05; **C** Representative microphotographs of tube formation by ECs; **D** Quantification of tube formation by ECs. n = 9, one-way ANOVA, Tukey multiple comparisons test. *p  < 0.05; ****p < 0.0001. HVF: high-power visual field

To obtain direct evidence that insufficient SOX6 results in EC dysfunction in IHL of db/db mice, we examined the effect of SOX6 overexpression on tube formation in diabetic ECs. Primary ECs were isolated from IHL of db/ + and db/db mice 3 days post-HLI and transfected with GFP-tagged SOX6 lentivirus or control lentivirus (CT, 10^7^ IU/ml) for 72 h. We observed that more than 95% mouse primary ECs were transfected with GFP-tagged SOX6 lentivirus (Fig. 8A). SOX6 mRNA expression was remarkably increased in mouse primary ECs transfected with SOX6 lentivirus (Fig. 8B). Tube formation activity by ECs from IHL of db/db mice was impaired compared to ECs from IHL of db/ + mice (Fig. 8C, D). Overexpression of SOX6 by transduction of SOX6 lentivirus dramatically improved tube formation by ECs from both db/ + and db/db mice (Fig. 8C, D). Our findings indicate that SOX6 is a critical target of miR-499-5p, and that suppression of SOX6 expression by miR-499-5p is responsible for EC dysfunction in IHL of db/db mice. Studies to determine if SOX6 overexpression in ECs can improve angiogenesis and hindlimb ischemic injury recovery in db/db mice are warranted.

## Discussion

In the present study, we provide multiple novel findings identifying a key role for SKMC-sEV-derived miR-499-5p in the diabetes/ischemia-impaired angiogenic property of ECs. First, we provide evidence that expression of muscle-specific miR-499-5p is enhanced in ECs from hindlimbs of db/db mice, and that enhanced miR-499-5p expression in the hindlimbs of db/db mice is further upregulated by ischemic injury. Second, sEVs from SKMC transfer miR-499-5p to ECs and targeted inhibition of either miR-499-5p or secretion of sEVs improves angiogenesis and blood perfusion in the IHL of db/db mice. Third, we provide evidence that miR-499-5p downregulates SOX6 to diminish the expression of multiple angiogenic factors. We demonstrate that targeting miR-499-5p-mediated pathogenic communication between SKMCs and ECs may be a novel avenue for therapy of CLI in patients with diabetes (**Graphical Abstract**).

miRNAs have been widely recognized as potent genetic regulators that influence diverse biological and developmental processes; thus, miRNAs play a pivotal role in the pathogenesis of various diseases. Previously, we reported that transient introduction of miR-294-3p in the heart promotes cardiomyocyte cell cycle reentry after ischemic injury [36]; negative regulation of miR-375 enhances bone marrow-derived progenitor cell-mediated myocardial repair and function after myocardial infarction; [16] and miR-375 attenuates post-myocardial infarction inflammatory response and left ventricular dysfunction [17]. Recently, we demonstrated that miR-331-5p in sEVs acts as a potent fibrotic repressor in heart failure [37]. Expression of miR-499-5p in circulating/tissue was regulated in patients/animals under ischemic and diabetic assault. Recently, miR-499-5p has been considered a risk factor of diabetes-mediated cardiovascular complication and a diagnostic marker for ischemic tissue injury, such as acute heart failure [38, 39]. However, a potential role for miR-499-5p in regulating EC function and angiogenesis has never been investigated particularly in the context of diabetes-induced vascular dysfunction, likely because miR-499 is expressed solely in muscle cells and not in ECs. Our study is the first reporting the critical role of muscle-specific miR-499-5p in impaired angiogenesis/neovascularization and IHL recovery in db/db mice. The results provide fundamental insight into therapeutic application of anti-miR-499-5p in improving CLI outcomes in patients with diabetes.

SKMCs have become an attractive target for the manipulation of therapeutic angiogenesis because they secrete either pro- or anti-angiogenic factors [11, 12, 40]. These secreted factors may promote cell–cell communication between SKMCs and ECs [11, 12, 40], either through direct cell–cell contact, transfer of secreted paracrine molecules, or exchange of extracellular vesicles [13]. Enhancing the paracrine function of SKMC is a potential strategy for therapy of IHL in experimental animals [14]. In diabetic patients/animal models, aberrant SKMC function may be involved in diabetes-impaired EC function/biology in ischemic limbs [11, 12]. Our findings that miR-499-5p expression was remarkably enhanced in the SKMCs and SKMC-derived sEVs, thus altering recipient EC function and ischemic limb neovascularization furthers the concept that muscle-EC communications play a central role in angiogenesis. Future studies to explore similar mechanisms in the context of other ischemic tissues, particularly in diseases such as myocardial infarction, could advance therapeutic treatment options on multiple fronts.

Small extracellular vesicles (sEVs), also called exosomes, are lipid bilayer membrane nanovesicles (diameter: 30–200 nm) carrying biologically active compounds (i.e., proteins, nucleic acids, miRNA, metabolites, and so on) [41]. sEVs secreted from their cellular source and eventually internalized by recipient cells may change the fate of recipient cells through autocrine or paracrine signaling in response to specific donor-cell exosome cargo [5]. sEVs have emerged as key paracrine regulators and therapeutic targets in cardiovascular diseases [25]. We have reported that sEVs secreted from healthy stem cells are beneficial for ischemic heart repair via improved viability, proliferation and tube formation of ECs [42]; enhancing neovascularization and cardiomyocyte survival, reducing inflammatory responses and fibrosis post-myocardial infarction (MI) [26, 27]. Recently, we reported that Tipifarnib, which targets three key proteins (Rab27a, nSMase2 and Alix) involved in biosynthesis and release of sEVs, remarkably improved heart function in the transverse aortic constriction mouse model [37]. We also observed that sEVs secreted from diabetic endothelial progenitor cells diminished the beneficial effects of healthy sEVs on ECs and cardiomyocytes in response to myocardial ischemia in db/db mice by mechanisms involving epigenetic alteration in recipient cells [28]. Our data showing that circulating sEVs from db/db mice negatively affects ischemic limb blood perfusion recovery in non-diabetic mice provide further evidence for the role of sEVs and their cargo in pathophysiology of cardiovascular diseases. Although sEVs contain multiple molecular regulators including signaling proteins metabolites and lipids, studies report that they also are highly enriched in non-coding RNAs, particularly miRNAs [43]. The role of EV-contained miRs has been extensively studied in cardiovascular disease. Our data in this study further establishes the role of sEV delivery of cell-specific miR in the functional alteration of recipient cells (that previously did not express or synthesize this miR) in the pathophysiology of disease. Our study suggests that sEV-derived miR-499-5p may be a biomarker for the severity and progression of CLI in patients with diabetes.

In *silico* analysis from miR target prediction programs, SOX6, a member of sex-determining region Y-box (SOX) transcription factor family, is one of the most likely genes targeted by miR-499-5p due to multiple binding sites in both human and murine SOX6 3’UTR (Fig. S6). In good accordance with previous studies [44, 45], we validated that overexpression of miR-499-5p decreases SOX6 protein and mRNA expression in ECs. Several members of the SOX family have been reported to regulate the angiogenic property of ECs under various pathophysiological conditions. Tube formation was promoted in by human umbilical vein ECs co-cultured with SOX5-overexpressed A549 cells (a human lung adenocarcinoma cell line) [35]. SOX-13 also showed to improve tube formation of glioma-exposed ECs [36]. SOX7/17 is expressed specifically in ECs and global deletion of SOX7/17 results in embryonic lethality with severely impaired angiogenesis in mice [33]. SOX18 is expressed transiently in ECs of developing blood vessels and capillaries in wounded skin [46, 47]. SOX*F* transcription factors consisting of SOX7, SOX17 and SOX18 were suggested to be indispensable players in developmental angiogenesis [33]. However, expression of SOX6 in ECs and a role of SOX6 in EC function with respect to diabetes has never been studied. In the present study, we not only identified SOX6 as a direct target of miR-499-5p but also established a key role of SOX6 in the regulation of EC function and angiogenic gene expression. Further experiments to explore the role of SOX6 and its downstream targets in the pathogenesis of IHL in diabetes are warranted to establish SOX6 as an independent regulator of angiogenesis, in vivo.

We acknowledge that our present studies did not explore certain additional aspects which may be addressed in future studies. Although the db/db mouse model has been extensively used as a type 2 diabetic animal model, there is still debate for using db/db mouse as type 2 diabetic animal model due to relevance of leptin receptor deficiency and the severity of phenotype representative of late-stage diabetes [48]. Therefore, it would be valuable to extend our study on another type 2 diabetes model with early-stage diabetes. High fat diet (HFD) is linked to an increased risk of type 2 diabetes and early-stages of type 2 diabetes [49]. Many factors are involved in the impairment of ischemic hindlimb recovery in mice fed with HFD [50], however, role of miR-499-5p in impaired blood perfusion and angiogenesis in response to ischemic hindlimb injury in HFD-induced type 2 diabetic mice remains unclear. As our limited data show that miR-499-5p was markedly increased in the skeletal muscle of HFD-induced diabetic mice (Fig. S2D), it would be interesting to evaluate if overexpressing miR-499-5p in skeletal muscles might show impaired angiogenesis/neovascularization and IHL injury in mice with early-stage diabetes or without diabetes. It needs to be noted that other contents carried by SKMC-sEVs modulated by diabetic conditions may also be synergistically involved in the pathogenesis of EC dysfunction in diabetic ischemic injury. Moreover, to validate the role of SKMC-sEVs in the pathogenesis of IHL injury in db/db mice, it would be worthwhile to examine the therapeutic effects of specific targeting on SKMC-sEVs biosynthesis when such tools become available. It is possible that miR-499-5p generated from diabetic SKMCs is transferred to ECs in a sEV-independent manner. Therefore, effects of specific targeting miR-499-5p in SKMCs may have additional therapeutic benefits which need to be investigated. Use of muscle specific sEV blockers, particularly those that inhibit not only release but also cargo sorting may be more potent alternatives than the GW4869 [51]. Moreover, in patient with diabetes, age is a significant factor impacting the risk and prognosis of CLI [52]. Previous studies suggested that effects/regulations of miR-499 and SOX6 in skeletal muscles may be age dependent [53, 54]. In this study, the role of miR-499-5p/SOX6 axis in diabetes/ischemia-impaired hindlimb injury recovery was limited in young db/db mice. Therefore, it may be worthy for future studies to explore if miR-499-5p/SOX6 axis in the pathogenesis of ischemic hindlimb injury in diabetes is age dependent.

In summary, we identified SKMC-sEVs-mediated miR-499-5p enhancement in ECs as a novel target causing EC dysfunction in IHL of db/db mice. miR-499-5p is packaged in SKMC-sEVs and transferred from SKMCs to ECs, where they suppress expression of transcription factor SOX6 thus decreasing generation/secretion of pro-angiogenic factors. Our studies reveal that targeting miR-499-5p-mediated pathogenic communication between SKMCs and ECs may be a novel avenue for angiogenic therapeutics of CLI in patients with diabetes.

## Supplementary Information

Additional file 1.

## Data Availability

No datasets were generated or analysed during the current study.

## Abbreviations

Ang 2: Angiopoietin-2
Anti-miR-Scr.: Anti-miR scramble
Anti-miR-499-5p: Anti-microRNA 499-5p
DAPI: 4′,6-Diamidino-2-phenylindole
EC: Endothelial cell
GM-CSF: Granulocyte-macrophage colony stimulating factor
HG: High glucose
IHL: Ischemic hindlimb
HMVECS: Human microvascular endothelial cell
HVF: High visual field
NC: Negative control
NG: Normal Glucose
miR: MicroRNA
miR-Scr.: MiR scramble
MyomiR: Muscle-specific microRNA
SKMC: Skeletal muscle cell
SKMC-sEV: Skeletal muscle-derived small extracellular vesicle
sEV: Small extracellular vesicle
SOX6: Sex-determining region Y-box 6
PDGF-AB/BB: Platelet-derived growth factor-AB/BB
uPA: Urokinase-type plasminogen activator
IGFBP-1: Insulin-like growth factor-binding protein
