# Supplementary material for: Muscle-specific miR-499-5p delivered by small extracellular vesicles impairs endothelial function and ischemic hindlimb recovery in diabetic mice
Source: Cardiovasc Diabetol. 2025 Jul 10;24:273. doi: 10.1186/s12933-025-02825-2 (PMC12243233; doi:10.1186/s12933-025-02825-2)
Supplement: Supplementary file 1 — Additional file 1. [file 12933_2025_2825_MOESM1_ESM.docx]

**SUPPLEMENTAL MATERIALS**

**Muscle-specific miR-499-5p delivered by small extracellular vesicles impairs endothelial function and ischemic hindlimb recovery in diabetic mice**

Cheng *et al*

**Running Title**: miR-499-5p in diabetes-impaired angiogenesis

**Address correspondence to:**

Raj Kishore, PhD

Aging + Cardiovascular Discovery Center

Lewis Katz School of Medicine

Temple University, 3500 Broad Street

Philadelphia, PA 19140, U.S.A

Phone: 215-707-2523

Fax: 215-707-9890

E-mail: [raj.kishore@temple.edu](mailto:raj.kishore@temple.edu)

Zhongjian Cheng, PhD

Aging + Cardiovascular Discovery Center

Lewis Katz School of Medicine

Temple University, 3500 Broad Street

Philadelphia, PA 19140, U.S.A

Phone: 215-707-2308

Fax: 215-707-9890

E-mail: [zjcheng@temple.edu](mailto:zjcheng@temple.edu)

**This file includes:**

Detailed Methods

Supplemental Figures and Figure Legends (S1-S6)

Supplemental Tables and Table Legends (Supplemental Table 1-3)

Supplemental References: 5, 6, 8, 30, 31, 32, 55

**Detailed Methods**

**Animal studies**

Male C57BL6J, db/+ and db/db mice at age of 8-10-week were obtained from Jackson Laboratories (Supplementary Table 1). All animal experiments were approved by the Institutional Animal Care and Use Committee of Temple University. Induction of ischemic hindlimb (IHL) in male C57BL/6J, db/+ or db/db mice was performed by ligation of left femoral artery as described previously.^5,6^ To study if silencing of miR-499-5p improves IHL repair in diabetes, we administered anti-miR-499-5p lentivirus by intramuscular injection of anti-miR-499-5p lentivirus (miRa-off-mmu-miR-499-5p lentivirus, Abm, LV002-c-53399, 5*10^7 IU in 100 µl PBS/mouse, at five injection sites, three sites in medial thigh and two sites in gastrocnemius muscles, respectively. 20 µl/per site) with a 27G needle as we previously described^5^) in db/db mice immediately after the left femoral artery ligation as we previously described.^5,6^ db/db mice treated with same dose of control lentivirus served as controls (Abm, m002). To examine whether SKMC-sEVs impaired blood perfusion recovery, we intramuscularly injected SMVC-sEVs (3*10^9 particles in 100 µl PBS, at five injection sites, three sites in medial thigh and two sites in gastrocnemius muscles, respectively. 20 µl/per site) with a 27G needle as we previously described^5^) from either db/+ or db/db mice into C57BL6J mice immediately after ligation of left femoral artery. C57BL6J mice injected with saline (vehicle) served as controls. To study the effects of blocking of sEV biosynthesis on IHL injury recovery, GW4689 (GW) was administered one week before IHL surgery (2 mg/kg bw, i.p., every other day) and continued up to 3 weeks after the surgery as described previously.^31^

**Laser Doppler perfusion imaging of hindlimb blood flow and sample collection**

Blood flow measurements in each mouse’s hindlimbs were performed on 37oC heated pads before (pre-) and immediately after surgery (day 0), and on days 3, 7, 14 and 21 post-ligation using a laser Doppler perfusion imager (model LDI2-IR, Moor Instruments, Wilmington, DE) as previously described.^5,6^ At each time point, an average of 4 measurements per animal was made on anesthetized (Avertin, i.p., 125 mg/kg) mice. To avoid the influence of light and temperature, the results were expressed as a ratio of perfusion in the left (ischemic) vs. right (non-ischemic) hindlimb.^5,6^ Perfusion was evaluated on the basis of colored histogram pixels and normalized for the limb surface analyzed. Blood perfusion in the IHL was calculated and presented as ratio of blood flow of ischemic to non-ischemic limb at time points or as area under carve (AUC) of ratio of IHL/non-IHL.^5^ In the end of experiments, gastrocnemius muscles from sham/ischemic hindlimb were dissected and fixed for immunostaining, or stored in -80oC for RT-PCR and Western blot, respectively.

**Necrosis score**

Tissue damage and hindlimb functionality were evaluated in db/db mice subjected to the IHL model with or without treatment of GW4869 on the 21st day postoperatively. The severity of necrosis was assessed using a scoring system, wherein a score of 0 denoted the absence of necrosis in the ischemic limb, a score of 1 indicated necrosis limited to the toe, a score of 2 represented necrosis extending to the dorsum of the foot, a score of 3 corresponded to necrosis extending to the crus, and a score of 4 indicated necrosis extending to the mid-tibia or complete limb necrosis.^32^

**Isolation and culture of mouse primary skeletal muscle cells**

Skeletal muscle cells (SKMCs) were isolated from skeletal muscles (SKMs) from hindlimb (HL), sham hindlimb (sham) or ischemic hindlimb (IHL) of wildtype, db/+ or db/db mice as previous described with modification (https://www.jove.com/video/50846/isolation-culture-and-transplantation-of-muscle-satellite-cells). Briefly, blood in SKMs was washed off by 1xPBS then connective tissue, blood vessels, nerve bundles and adipogenic tissue were removed under dissection microscope. The tissue was cut into small pieces in DMEM then incubated in collagenase I solution (500 µg/ml in serum-free DMEM, Sigma, C5894-50MG) at 37oC for 30 minutes with vertexes (≈10 seconds) each 10 minutes. Then added the same volume of 10% FBS DMEM to stop the digestion. The cellular digests were filtered through 70 μm and 40 μm cell strainers and centrifuged at 2,000 rpm at 4oC for 5 min. The supernatant was aspirated and discarded. Cells were washed with 10% FBS DMEM for three times (2,000 rpm at 4oC for 5 min). The cells were pre-plated in non-coated 10 cm dishes in skeletal muscle cell growth medium (10^6 cells/dish, Lonza, CC3245) for 45 minutes. SKMCs in the supernatant medium were seeded to 0.2% gelatin coated 10 cm dish/mouse and cultured, SKMCs isolated from db/db mice were cultured in high glucose condition ( SkGM-2 Skeletal Muscle Cell Growth Medium-2 BulletKit, CC-3245, LonzaTM, plus 25 mM additional D-glucose ), whereas SKMCs isolated from db/+ or wildtype (C57BL/6J) mice were cultured without addition of 25 mM D-glucose for 7-10 days before processing next experiment. SKMCs were identified and analyzed for purification by immunostaining with desmin antibody (PA5-17182, Thermo Fisher, Fig. S1).

**Isolation and culture of mouse primary endothelial cells**

Endothelial cells (ECs) from skeletal muscles on HL, sham and IHL of wildtype, db/+ or db/db mice were isolated by collagenase I and CD31 Dynabeads and cultured as described previously.^5,6,8,55^ ECs isolated from db/db mice were cultured in high glucose condition (EGM-2 MV BulletKit, CC-3156 & CC-4147, LonzaTM, plus 25 mM additional D-glucose), whereas ECs isolated from db/+ or wildtype (C57BL/6J) mice were cultured without addition of 25 mM D-glucose for 10-14 days before processing next experiment.

**ECs treatment with sEVs**

To study the effect of diabetic SKMC-sEVs on EC biology/function, human cardiac microvascular endothelial cells (HMVECs, Cat. #: CC-7030. Lonza) were treated with SKMC-sEVs collected from either male db/+ or db/db mice at age of 8-10-week (10^8 particles/ml, 48 hrs) in sEV-free culture medium (EBM-2, Cat. #: CC-3156 & CC-4147 excluded FBS, 10% sEV-free FBS, Lonza) for 48 hrs after 6 hrs starvation (1% FBS).

**Treatment of mouse primary SKMCs with high glucose**

To study effect of hyperglycemia, the key effector responsible to pathogenesis of cardiovascular diseases in diabetes, on miR-499-5p expression, we treated the SKMCs isolated from C57BL6J mice with high glucose (HG) by adding 25 mM D-glucose (D-Glu.) to culture medium after 6 hrs starvation (1% FBS). Cells in the culture medium with 25 mM mannitol (osmotic control, 5 mM D-glucose, OC) or without addition of D-glucose (normal glucose, 5 mM D-glucose, NG) served as controls.

**Isolation of sEVs**

Serum-derived sEVs (serum-sEVs) and SKMC-derived sEVs (SKMC-sEVs) were isolated from serum and sEV-free FBS culture medium of SKMCs by ultra-centrifugation as we described previously.^6^ Culture medium of the primary SKMCs was replaced with 10% sEV-free FBS culture medium of SKMCs 4 days post-isolation. The sEV-free FBS culture medium was collected after 3 days. The serum and sEV-free FBS culture medium were clarified by centrifugation (14.000g for 20 minutes) then collected by ultra-centrifugation (100,000g for 1 hr) on 30% sucrose-D2O solution (density ~1.127 g/cm3), washed with 1xPBS and pelleted. The purified sEV fractions were re-suspended in 1xPBS for usage. Quality/quantity of sEVs was evaluated by Nano-sight instrument (NS300).

**Oligonucleotide transfection**

To study the role of miR-499-5p in biology/function of ECs, Cells were transfected in SKMCs/ECs with miR-499-5p mimics (Cat. #: MC11352, 20 ng/ml, Thermo Fisher) or inhibitor (Cat. #: MH11352, 50 ng/ml, Thermo Fisher) plus lipofectamine (3 μl/ml, Cat. #: 13778030, BD, Supplementary Table 2) for 72 hrs. miRNA mimics- and inhibitor-scramble (Cat. #: 4464058 and 4464076, respectively, Thermo Fisher) were served as controls. Efficiency of transfection was examined by RT-PCR using miR-499-5p primer (Cat. #: 1352, Thermo Fisher).

**siRNA transfection**

To study the role of SOX6 in the regulation of biology/function of ECs, HMVECs were transfected with SOX6 small interfering RNA (SOX6 siRNA, Cat. #: 133643, ThermoFisher, 200 nM) plus lipofectamine (3 μl/ml, Cat. #: 13778030, BD) for 72 hrs according to manufacturers’ protocol. HMVECs transfected with negative control siRNA (NC, Silencer@Negative Control #1 siRNA, Cat. #: AM4611, ThermoFisher) served as controls. The efficiency of transfection was examined by RT-PCR using SOX6 primer (Supplementary Table 2).

**Transduction of SOX6 lentivirus in mouse primary ECs isolated from IHL**

GFP-tagged mouse SOX6 plasmid (MR227129L4, Origene) was amplified by transformation of plasmid DNA into E coli using the heat shock methods and packaged into lentivirus by Dr. Sudarsan Rajan in-house. To study the role of SOX6 in diabetes-impaired EC function in db/db mice, mouse primary ECs isolated from IHL skeletal muscles of db/+ and db/db mice (3 days post-IHL) were transduced with GFP-tagged SOX6 lentivirus (10^7 IU/ml) for 72 hrs. ECs from IHL of db/+ and db/db mice treated with GFP-tagged control (CT) lentivirus served as controls. Tube formation was processed for examining the effect of overexpression of SOX6 on EC function in IHL of db/db mice. Efficiency of lentivirus transduction was examined with GFP positive under florescent microscope and SOX6 mRNA expression by RT-PCR.

**Co-culture of SKMCs and ECs**

SKMCs and HMVECs (100,000 cells/per well for both cell types) were seeded into the well inserts (upper chambers) and plates (lower chambers), respectively. HMVECs were cultured with 10% sEV-free FBS culture medium (EBM-2, Cat. #: CC-3156 plus CC-4147 excluding FBS, Lonza). Well inserts for 6-well plates with a 0.4 µm-pore filter were used following the manufacturer's instruction (Corning Incorporated, Costar, Cat. #07-200-165). To examine whether diabetic SKMC-sEVs can act as vehicles to transfer miR-499-5p cargo from SKMCs to ECs thereby impair EC function/biology, we examined miR-499-5p levels in ECs co-cultured with either diabetic or non-diabetic SKMCs in the presence or absence of sEV inhibitor GW (1, 5 or 10 µM) for 48 hrs. SKMCs isolated from db/db mice were cultured in high glucose condition ( SkGM-2 Skeletal Muscle Cell Growth Medium-2 BulletKit, CC-3245, LonzaTM, plus 25 mM additional D-glucose ), whereas SKMCs isolated from db/+ or wildtype (C57BL/6J) mice were cultured without addition of 25 mM D-glucose. To provide direct evidence that miR-499-5p-overexpressed SKMCs can transfer miR-499-5p cargo from SKMCs to ECs, SKMCs isolated from wildtype mice was transfected with miR-499-5p mimics or miR scramble (Scr.) for 72 hrs, then cultured in the upper channel in the presence or absence of GW (5 or 10 µM) for 48 hrs. In the end of experiments, HMVECs were collected for examining miR-499-5p levels by RT-PCR.

**Tube formation**

Tube formation by ECs was performed as we described previously.^5,6^ Briefly, 4x104 ECs were plated on 120 µl or 60 µl Matrigel (BD Falcon) in a 48-well or 96-well plate, respectively. After incubation at 37oC in an atmosphere of 5% CO2 for 16 hrs, the tubes were observed and photographed using a high-power phase contrast microscope (Nikon, TS100). The tube formation was counted by number of branches in 5 random/high-power vision fields (HVF) using ImageJ.

**Migratory activity**

Migratory activity of ECs was performed using a modified Boyden chamber (Coning, Cat. #: 3422) as described previously.^30^ Briefly, we placed 100 µl basal medium (EBM-2, Cat. #: CC-3156, Lonza) in the upper chamber and 750 µl culture medium (EBM-2 containing EGM-2mv, Cat. #: CC-4147, Lonza) and EGM-2 SignleQuot bullet kit Suppl & Growth Factors (Cat. #: CC-4176, Lonza). HMVECs (10^5 cells/well) were suspended in 200 µl basal medium then placed in the upper chamber. After 16 hrs incubation, the cells on the upper surface were removed and the cells on the underside were fixed with 3.7% formaldehyde and stained with Giemsa. The magnitude of EC migration was evaluated by counting the migrated cells in 5 random/high-power vision fields (HVF) by ImageJ.

Immunohistochemistry

Muscle sections (5 µm thickness) were heated with citrate buffer at 95°C for 40 minutes for antigen retrieval. After blocking with 5% BSA in phosphate-buffered saline (PBS), muscle sections were co-incubated with polyclonal goat antibody against CD31 (EC marker, 1:30, Cat. #: AF3628, R&D) and monoclonal mouse antibody against α-smooth muscle actin (α-SMA, smooth muscle cell marker 1:1000, Cat. #: A2547, Sigma) overnight at 4°C. For every section, negative control without primary antibody was processed simultaneously. After 15 minutes of washing with 1xPBS, secondary antibodies were added for 1 hrs at room temperature (1:200). Cell nuclei were counter-stained with DAPI (Molecular Probes). CD31 and α-SMA were stained with fluorescent secondary antibody against mouse (red) and rabbit (green), respectively. The capillary and arteriole density were then determined by counting the density of capillaries (CD31+) and arterioles (α-SMA+) in each section of muscle. We also examined SOX6 expression in primary ECs isolated from IHL of db/+ and db/db mice days post-IHL with polyclonal rabbit SOX6 (1:200, Cat. #: 30455, Abcam). Images were taken by a fluorescent microscope (Nikon TIE3000) for CD31/α-SMA/DAPI and Leica SP8 confocal microscope (Leica Micro system, Inc) for SOX6/DAPI imaging, respectively. CD31, α-SMA and SOX6 density was counted by ImageJ.

**Angiogenic protein profile array**

To explore the role of SOX6 in secretion of angiogenic factors in ECs, we examined angiogenic factors in condition culture medium of HMVECs transfected with either SOX6 or negative control siRNA for 72 hrs using Proteome Profiler Human Angiogenesis Array Kit (Cat. #: ARY007, R&D System) according to the manufacturer’s protocol. Quantification of selected angiogenic factors was conducted by densitometry using ImageJ.

**RT–PCR**

RNA was collected with Trizol RNA isolation kit then reverse transcribed with iScript cDNA Synthesis Kit (Bio-Rad Laboratories, Hercules, CA) or miRNeasy Mini kit (Cat. #: 217004, Giagen, USA) for mRNAs and miRs, respectively. Expression of different miR gene was measured using quantitative miRNA stem loop RT–PCR technology (TaqMan miRNA Assays; Applied Biosystems). This assay uses gene specific stem cell loop RT primers and TaqMan probes to detect mature miRNA transcripts. Transcription was performed using 10-50 ng total RNA and TaqMan miRNA RT kit (Applied Biosystems). For measuring of expression of mRNA, amplification was performed using a SYBR Green (Applied Biosystems, Foster City, CA). The primer sequences were synthesized by IDT as shown in Supplementary Table 2. Relative miR and mRNA expression of target genes was normalized to the endogenous housekeeping gene U6 and GAPDH, respectively.

Western blot

Protein extracted from the ECs (10 μg) was used for fractionation by 9% SDS-PAGE and transferred to nitrocellulose blot. SOX6 expression was detected by using rabbit polyclone SOX6 antibody (1:1000, ab30455, Abcam, Supplementary Table 3) and respective second antibody. Signals were detected and quantified using Odyssey® Fc Imaging System (LI-COR Biosciences, model number 2800).

Chemicals

GW4869 (Cat. #: 13127, Cayman) was diluted by DMSO (1 µM, final concentration of DMSO was 0.1%). ECs treated with 0.1% DMSO (vehicle, Vehi.) served as controls. If not otherwise specified, all chemicals were purchased from Sigma-Aldrich.

**Supplemental Figures and Figure Legends**


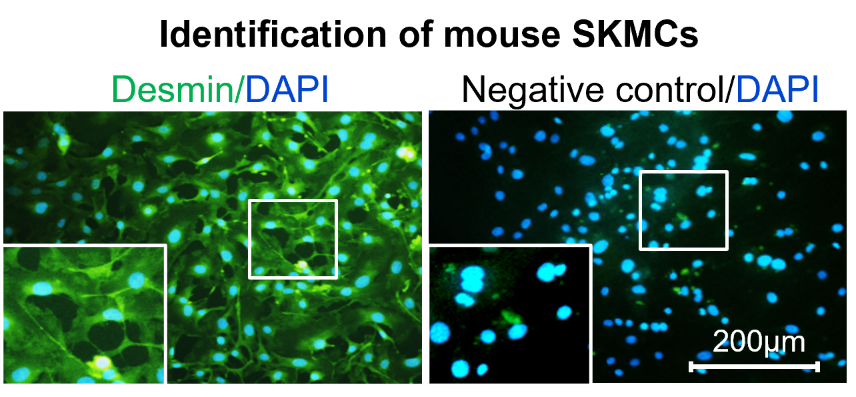


**Fig. S1. Identification of mouse primary skeletal muscle cell (SKMC).** SKMCs were isolated from mouse hindlimbs and identified by immunostaining of muscle marker desmin (green). SKMC nuclei were stained with 4′,6-diamidino-2-phenylindole (DAPI, blue).

**
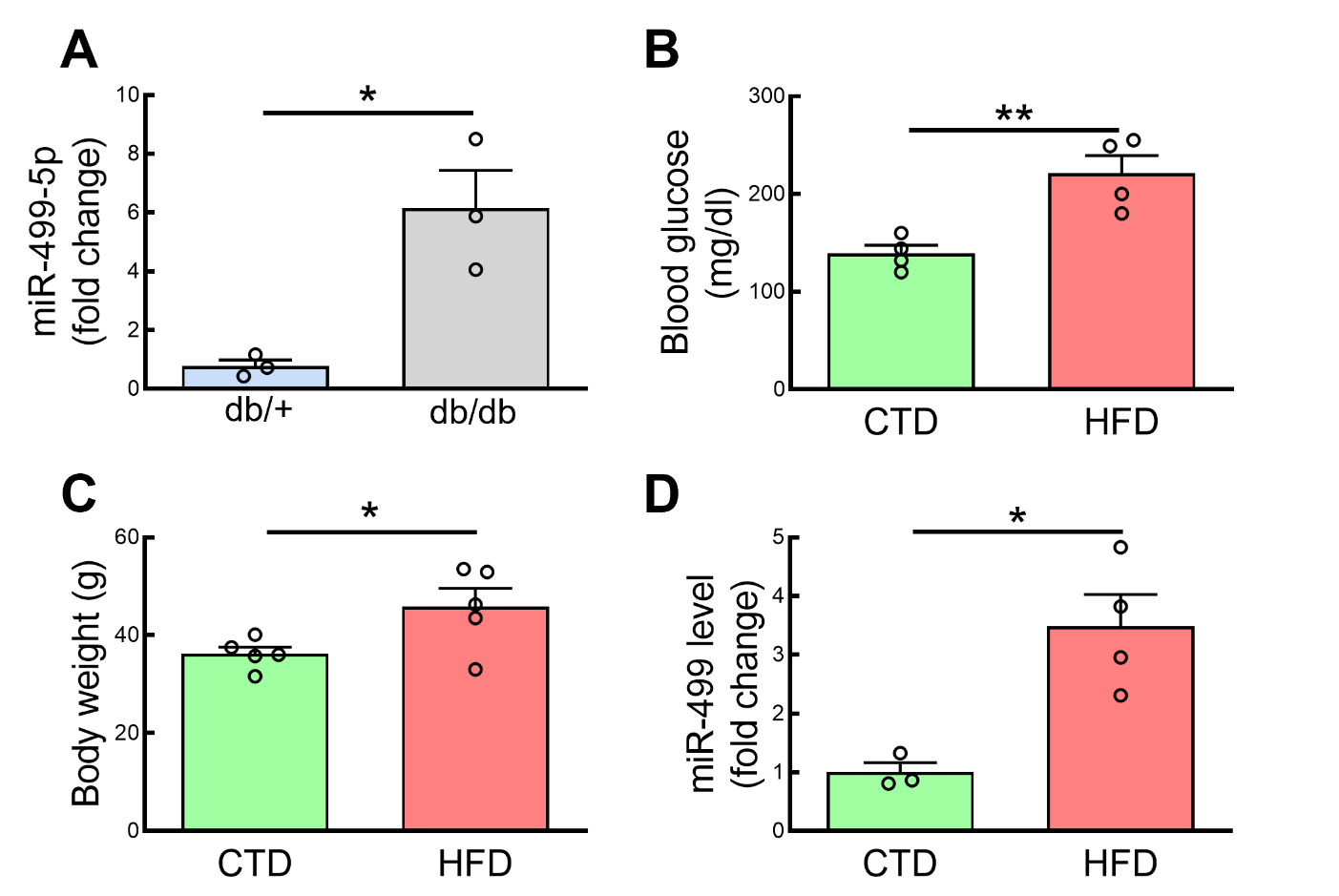
**

**Fig. S2. miR-499-5p expression is increased in skeletal muscles of diabetic mice.** (A). miR-499-5p expression in skeletal muscles of db/+ and db/db mice. Gastrocnemius muscles were isolated from hindlimbs of male db/+ and db/db mice at age of 8 weeks for miR-499-5p levels determination by PCR. (B). Blood glucose levels in high fat diet-induced diabetic mice; (C). Body weights of high fat diet-induced diabetic mice; (D). miR-499-5p expression in skeletal muscles of high fat diet-induced diabetic mice. Eight-week-old-male C57BL/6J mice were fed with high fat diet (HFD, Research Diet, D12492) or control diet (CTD, 12450J) for 14 weeks. At the end of the experiments, body weight was measured, and gastrocnemius muscles were collected from hindlimbs of mice for determination of miR-499-5p levels by PCR. Blood glucose levels were determined using OneTouch Ultra Test Strips (OneTouch). n=3-5, unpaired student *t* test. *p<0.05; **p<0.01.


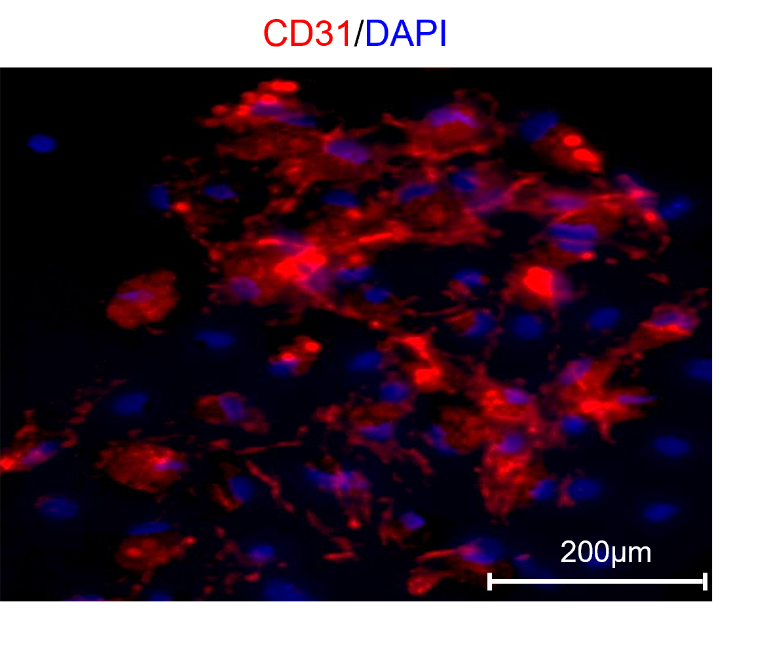


**Fig. S3. Identification of mouse primary endothelial cell (EC).** ECs were isolated from mouse hindlimbs and identified by immunostaining of EC marker CD31 (red). Nuclei were stained with 4′,6-diamidino-2-phenylindole (DAPI, blue).


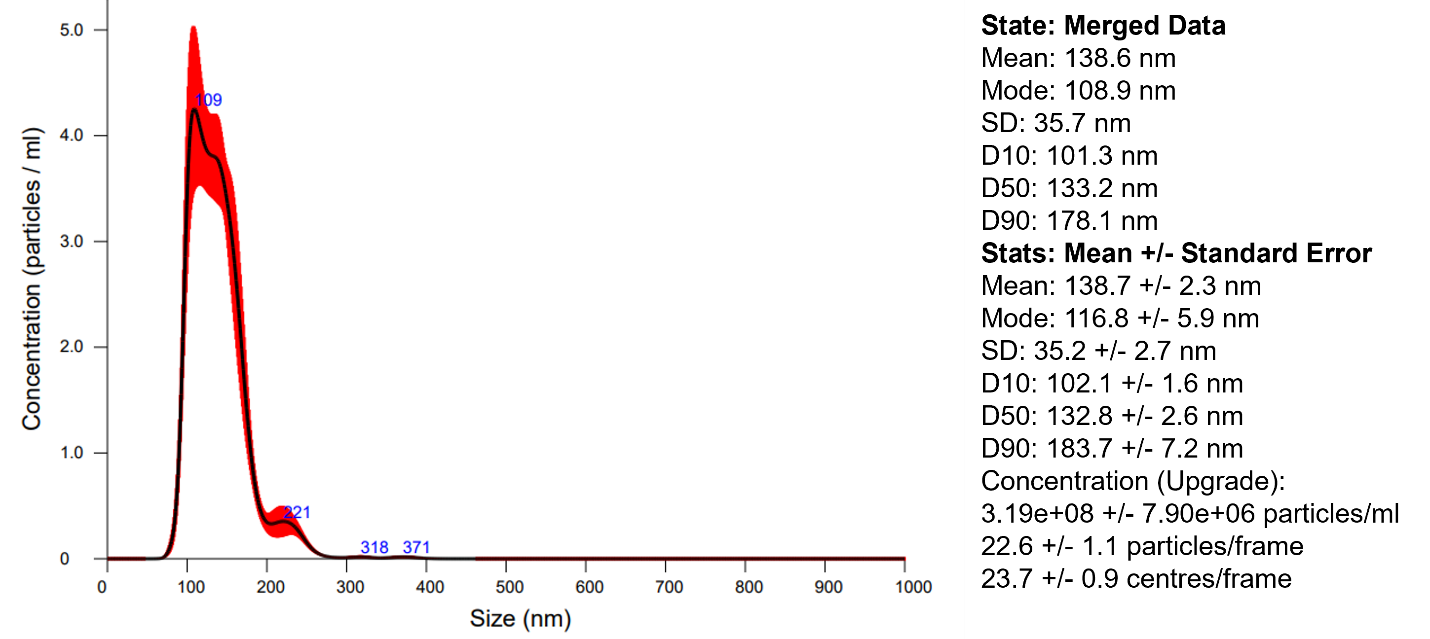
**Fig. S4. Representative characterization of the isolated mouse SKMC-sEVs.** Mouse SKMC-sEVs were isolated from culture medium of SKMCs by ultra-centrifugations. Size and concentration of SKMC-sEVs was evaluated by Nano-sight analysis (NS300).


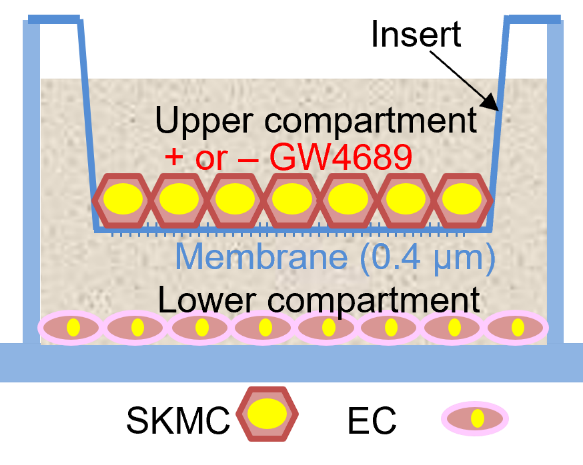


**Fig. S5. A scheme of cell co-culture system.** ECs were cultured in the lower chamber and mouse SKMCs were cultured in the upper chamber (insert) in the presence or absence of GW4869 (1, 5 or 10 µM) for 48 hrs. Both chambers were pre-coated with 0.2% gelatin.


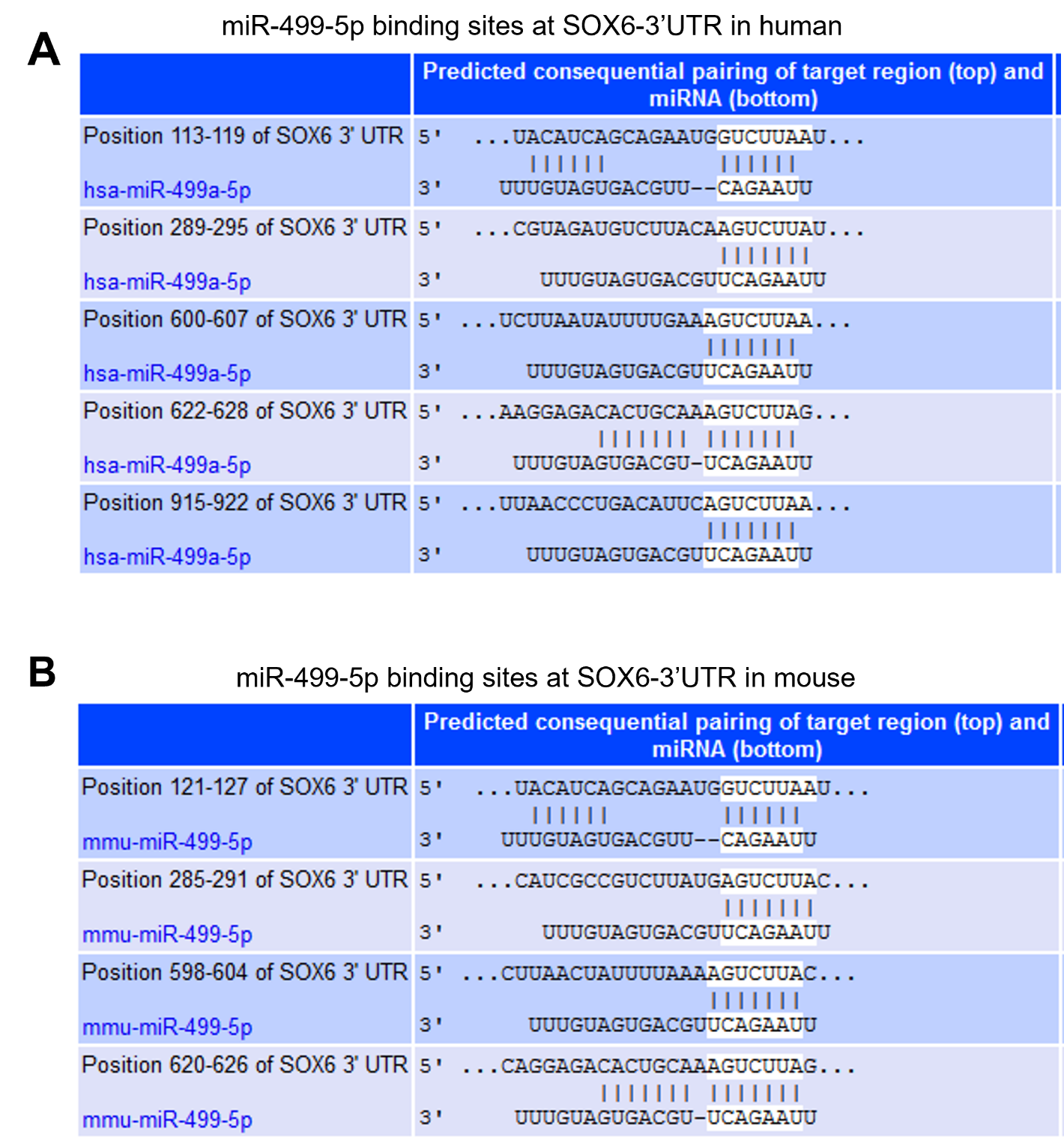


**Fig. S6. miR-499-5p has multiple binding sites at SOX6-3’UTR in human (A) and mouse (B).**

| **Supplemental Figures and Figure Legends**  **Supplementary Table 1: Animals (in vivo and in vitro studies)** | | | | | | | | | |  |  |
| --- | --- | --- | --- | --- | --- | --- | --- | --- | --- | --- | --- |
| **Species** | **Vendor or Source** | | | **Strain/Background** | | | **Sex** | **Persistent ID/URL** | | | |
| Mouse | Jackson Labs | | | C57BL/6J | | | Male | [000664 - B6 Strain Details](https://www.jax.org/strain/000664) | | | |
| Mouse | Jackson Labs | | | db/+ /Heterozygous for Dock7m Heterozygous for Leprdb | | | Male | [000642 - Strain Details](https://www.jax.org/strain/000642) | | | |
| Mouse | Jackson Labs | | | db/db/Wildtype for Dock7m, Homozygous for Leprdb | | | Male | [000642 - Strain Details](https://www.jax.org/strain/000642) | | | |
| **Supplementary Table 2: Sequences of PCR primers, miRNA mimics and antagomirs** | | | | | | | | | | |  |
| **Items** | | **Species** | **Assay Name** | | **Assay ID** | **miRbase ID** | | | **Mature miRNA Sequence** | |  |
| miR-499-5p mimics | | mouse & human | hsa-miR-499a-5p | | MC11352 | hsa-miR-499a-5p (human); mmu-miR-499-5p (mouse) | | | UUAAGACUUGCAGUGAUGUUU | |  |
| miR-499-5p inhibitor | | mouse & human | hsa-miR-499a-5p | | MH11352 | hsa-miR-499a-5p (human); mmu-miR-499-5p (mouse) | | | UUAAGACUUGCAGUGAUGUUU | |  |
| miR-499-5p primer (TaqMan^TM^ MicroRNA Assay) | | mouse & human | mmu-miR-499 | | 001352 | hsa-miR-499a-5p (human); mmu-miR-499-5p (mouse) | | | UUAAGACUUGCAGUGAUGUUU | |  |
| miR-499-3p primer (TaqManTM MicroRNA Assay) | | mouse | mmu-miR-499 | | 464040_mat | mmu-miR-499-3p | | | GAACAUCACAGCAAGUCUGUGCU | |  |
| **Items** | | **Species** | **Assay Name** | | **Assay ID** | **Forward primers** | | | **Reverse primers** | |  |
| SOX6 primer | | mouse | N/A | | N/A | GGTCATGTTTCCCACCCACAA | | | TTCAGAGGGGTCCAAATTCCT | |  |

**Supplementary Table 3: Antibodies**

| **Target antigen** | **Vendor or Source** | **Catalog #** | **Working concentration** | **Persistent ID / URL** |
| --- | --- | --- | --- | --- |
| Mouse | abcam | Ab30455 | 1:1000 | [Anti-SOX6 antibody. Rabbit polyclonal (ab30455) \| Abcam](https://www.abcam.com/en-us/products/primary-antibodies/sox6-antibody-ab30455?srsltid=AfmBOoppMBrAze7_1MxLUmDidy75AeXEzmoazxgg3E80YRIqbBKxNJxm) |
